# Exploring eHealth Literacy and Patient-Reported Experiences With Outpatient Care in the Hungarian General Adult Population: Cross-Sectional Study

**DOI:** 10.2196/19013

**Published:** 2020-08-11

**Authors:** Zsombor Zrubka, Óscar Brito Fernandes, Petra Baji, Ottó Hajdu, Levente Kovacs, Dionne Kringos, Niek Klazinga, László Gulácsi, Valentin Brodszky, Fanni Rencz, Márta Péntek

**Affiliations:** 1 Department of Health Economics Corvinus University of Budapest Budapest Hungary; 2 University Research and Innovation Center Óbuda University Budapest Hungary; 3 Department of Public and Occupational Health, Amsterdam UMC Amsterdam Public Health Research Institute University of Amsterdam Amsterdam Netherlands; 4 Department of Comparative Economics Eötvös Loránd University Budapest Hungary; 5 Physiological Controls Research Center University Research and Innovation Center Óbuda University Budapest Hungary; 6 Premium Postdoctoral Research Program Hungarian Academy of Sciences Budapest Hungary

**Keywords:** health literacy, eHealth literacy, patient-reported experience measures, patient-reported outcome measures, ambulatory care, shared decision making, Hungary, survey

## Abstract

**Background:**

Digital health, which encompasses the use of information and communications technology in support of health, is a key driving force behind the cultural transformation of medicine toward people-centeredness. Thus, eHealth literacy, assisted by innovative digital health solutions, may support better experiences of care.

**Objective:**

The purpose of this study is to explore the relationship between eHealth literacy and patient-reported experience measures (PREMs) among users of outpatient care in Hungary.

**Methods:**

In early 2019, we conducted a cross-sectional survey on a large representative online sample recruited from the Hungarian general population. eHealth literacy was measured with the eHealth Literacy Scale (eHEALS). PREMs with outpatient care were measured with a set of questions recommended by the Organisation for Economic Co-operation and Development (OECD) for respondents who attended outpatient visit within 12 months preceding the survey. Bivariate relationships were explored via polychoric correlation, the Kruskal–Wallis test, and chi-square test. To capture nonlinear associations, after controlling covariates, we analyzed the relationship between eHEALS quartiles and PREMs using multivariate probit, ordinary least squares, ordered logit, and logistic regression models.

**Results:**

From 1000 survey respondents, 666 individuals (364 females, 54.7%) were included in the study with mean age of 48.9 (SD 17.6) years and mean eHEALS score of 29.3 (SD 4.9). Respondents with higher eHEALS scores were more likely to understand the health care professionals’ (HCPs’) explanations (*χ*^2^_9_=24.2, *P*=.002) and to be involved in decision making about care and treatment (*χ*^2^_9_=18.2, *P*=.03). In multivariate regression, respondents with lowest (first quartile) and moderately high (third quartile) eHEALS scores differed significantly, where the latter were more likely to have an overall positive experience (*P*=.02) and experience fewer problems (*P*=.02). In addition, those respondents had better experiences in terms of how easy it was to understand the HCPs’ explanations (*P*<.001) and being able to ask questions during their last consultation (*P*=.04). Patient-reported experiences of individuals with highest (fourth quartile) and lowest (first quartile) eHEALS levels did not differ significantly in any items of the PREM instrument, and neither did composite PREM scores generated from the PREM items (*P*>.05 in all models).

**Conclusions:**

We demonstrated the association between eHealth literacy and PREMs. The potential patient-, physician-, and system-related factors explaining the negative experiences among people with highest levels of eHealth literacy warrant further investigation, which may contribute to the development of efficient eHealth literacy interventions. Further research is needed to establish causal relationship between eHealth literacy and patient-reported experiences.

## Introduction

People-centeredness has shaped the cultural transformation of medicine, where we transitioned from a traditional paternalistic model toward a new model of care, grounded in partnerships and putting patients’ values and preferences in the forefront of medical decision making [1,2]. To cope with the ever-increasing pressure on health care budgets [3], improving the technical efficiency of health care systems remains a key imperative, in which eliminating waste and maximizing the value that matters to patients is a top priority for developed economies [4-7]. Therefore, in addition to its humanistic merits, people-centeredness has a strong economic rationale: enhancing the participation of patients in the health production process [4-8]. Hence, to involve people in health production and operationalize people-centered care, it is necessary that people have the information, education, and support they need to inform decision making about their own care [8].

Digital health is a key driving force behind the cultural transformation of medicine toward people-centeredness [9,10]. Digital health encompasses the use of information and communications technology in support of health and health-related fields (electronic health [eHealth]) and emerging areas such as the use of computing sciences in big data, artificial intelligence, and genomics [11,12]. Currently, the implementation of digital tools that facilitate people-centered care is a priority for policymakers [13]. With the digital transformation of health care, people have easier access to health information from online sources; simultaneously, people are called to assume responsibility for evaluating the accuracy and reliability of such information, and thus, rely on their eHealth literacy. eHealth literacy has been defined as “the ability to seek, find, understand, and appraise health information from electronic sources and apply the knowledge gained to addressing or solving a health problem” [14]. Higher levels of eHealth literacy have been associated with better subjective health status [15-17], healthier lifestyle [18-21], and lower risk of chronic disease [22]. Furthermore, with appropriate interventions eHealth literacy has been shown to be a modifiable factor even in later stages of life [23-25]. With the spread of people-centric values, patient-reported experience measures (PREMs)—quality indicators of health care from the patients’ perspective—have gained international attention. Signaling this is the work developed by the Organisation for Economic Co-operation and Development (OECD), where PREMs are used to evaluate the performance of health systems through a patient’s experience of care, including that of the patient–physician relationship [26].

Hungary has a tax-funded single-payer health system providing universal health coverage for the population. Most inpatient and specialist ambulatory care services are delivered by the public health system. Primary care—provided by private general practitioners (GPs)—acts as a gatekeeper. Per-capita spending on health care is among the lowest and the share of out-of-pocket contributions including informal payments is among the highest within the European Union. Life expectancy lags behind most European Union countries, mainly driven by lifestyle-related causes. Health inequalities are largely determined by sociodemographic variables [27-29]. Recent evidence suggested the need for improving patients' experience of care in Hungarian outpatient settings with regard to shared decision making [30]. This is aligned with findings of another study, which showed that patients’ preferences and interests were less likely to be taken into consideration by GPs in Hungary, in comparison with other European countries [29].

By making inferences from traditional health literacy studies to the eHealth domain [31], one may assume that higher eHealth literacy enables patients to orientate better in the health care system, find better access to care, use online information efficiently to reduce waiting times, and make more out of the interaction with health care professionals (HCPs). Among others, these factors may ultimately lead to better overall experience. Online health information may enhance the patient–physician relationship and although the evidence is mixed [32,33], the importance of eHealth literacy in translating the benefits of innovative digital health solutions to better experiences of care has been recognized [34-36]. For example, positive relationship between eHealth literacy and shared decision making has been found [37] and its contribution to patients’ decision-making styles has been demonstrated [32]. However, to our knowledge, no studies have focused on the relationship between eHealth literacy and PREMs to date.

This study aims to explore the association between eHealth literacy and OECD’s set of recommend PREMs for users of outpatient care, who were recruited from a large representative online sample from the Hungarian general adult population.

## Methods

### Study Design and Sample

We considered the CHERRIES checklist when reporting this study [38]. In early 2019, we conducted a large cross-sectional interned-based survey among the general adult population of Hungary, which explored eHealth literacy [39]. In addition, shared decision making [40] and PREMs [30,41] were investigated among those respondents, who used ambulatory care over the past 12 months due to health problems. We recruited 1000 online respondents. Quotas were used to ensure the representativeness of the sample according to the 2011 Population Census [42] data by gender, age, educational level, type of settlement, and NUTS 1 (Nomenclature of Territorial Units for Statistics) region of residence, including a fair representation of people aged 65 and over. Recruitment and data collection were carried out from a commercial online panel by a survey company (Big Data Scientist Kft); reports on dropout rates and the sampling frame were not available. All materials were in Hungarian and all participants spoke the same language (Hungarian). Participation was voluntary and anonymous. No incentives were offered for answering the survey. Participants gave their informed consent prior to the study. Ethical approval was obtained from the Hungarian Medical Research Council (ID: 47654-2/2018/EKU). The electronic questionnaire was piloted by the authors. Respondents could revise and change their answers for completed items, and full completion was required unless the “do not know/do not want to answer” option was offered.

Our sample included those respondents who had a face-to-face appointment with an HCP in the previous 12 months due to their own health problems and answered whether or not the visit had happened at their usual HCP.

### eHealth Literacy Scale (eHEALS)

eHealth literacy was measured with the Hungarian version of the self-reported eHealth Literacy Scale (eHEALS) [39,43]. This instrument has been used internationally both as a descriptive tool [22,39] and as a patient-reported outcome measure of digital health interventions [19,23,43]. The eHEALS consists of 8 items, each scored on a 5-point Likert scale. Items 1 and 2 are related to the awareness of health resources (“I know what health resources are available on the Internet”; “I know where to find helpful health resources on the Internet”); items 3 and 4 are related to searching for health resources (“I know how to find helpful health resources on the Internet”; “I know how to use the Internet to answer my questions about health”); items 5 and 8 are related to the utilization of health resources (“I know how to use the health information I find on the Internet to help me”; “I feel confident in using information from the Internet to make health decisions”); and items 6 and 7 are related to the appraisal of health resources (“I have the skills I need to evaluate the health resources I find on the Internet”; “I can tell high quality health resources from low quality health resources on the Internet”). Item levels are added for a total score ranging from 8 to 40. Higher scores indicate greater eHealth literacy [43]. The psychometric properties of the Hungarian eHEALS as well as its association with health outcomes and behavioral health risk factors in the general population have been shown in the validation study [39]. Because eHEALS showed convex relationship with self-rated health in the validation study, we decided to group respondents into 4 quartiles to explore potential nonlinear associations with PREMs in an easily interpretable manner [39,44,45]. We also performed sensitivity analysis using alternative eHEALS category boundaries. The eHEALS questionnaire is included in Multimedia Appendix 1.

### OECD-Proposed Set of Questions on Patients’ Experiences with Ambulatory Care (PREM)

Respondents’ experiences with ambulatory care were assessed by the set of questions recommended by the OECD’s Health Care Quality Indicators Project [26]. The questionnaire consists of 2 sections: (1) access to care, including questions on unmet medical needs and waiting times; and (2) patient experiences. The *access to care* section consists of 4 binary questions about unmet medical needs over the past 12 months (missed medical visits, interventions, or medications due to travel difficulties, or cost burden) and 4 questions on waiting times concerning the last medical appointment: waiting time to get the appointment (appointment waiting time) and on the day of consultation (office waiting time), and whether waiting was a problem in either case. In the *patient experiences* section, respondents were asked if the HCP (1) spent enough time with them; (2) provided easy-to-understand explanations; (3) provided the opportunity to ask questions or raise concerns about recommended treatment; and (4) involved in decisions about care and treatment as much as the respondent wanted to be. Answers were recorded on a 4-point Likert scale, with higher scores indicating more perceived problems. In the final item, respondents rated their perception of the overall quality of the appointment on a 5-point Likert scale ranging from *poor* (0) to *excellent* (4). Additional items inquired the HCP type, setting and time of the last visit, and whether the respondent visited his/her usual HCP. Questions related to unmet medical needs were posed if respondents had had health problems over 12 months preceding the survey, while waiting times and patient experiences were inquired only if the respondent had participated in ambulatory consultation with an HCP. The full PREM questionnaire is included in Multimedia Appendix 2.

### PREM Scores

Following the practice of countries using PREMs for monitoring health system performance, we created composite scores from PREM items [26]. The *Unmet Medical Needs Score* (range 0-4) reflected the number of areas where respondents experienced an unmet need (missed visit due to travel burden; missed visit due to cost burden; missed intervention due to cost burden; and missed medication due to cost burden). The binary *Any Unmet Medical Need* variable indicated if patients experienced unmet need in any of the 4 items. Waiting times were transformed to continuous variables by considering the midpoint of the respective waiting time answer option. Log-waiting times were used in regression analyses, assuming a 0.5-day waiting time for respondents with an appointment on the same day. We used the binary variable *Any Waiting Problem* to indicate if the office waiting time or appointment waiting time was a problem to the respondent. The 4 PREMs were used to create a composite *Problem Score*, which was the sum of the individual answer options of each PREM (1=yes, definitely; 2=yes, to some extent; 3=no, not really; and 4=definitely not). Hence, the composite score ranged from 4 to 16, where higher values represent more problems experienced during the visit. We also constructed a *Negative Experiences Score* (range 0-4) by counting the PREM items that did not receive a “yes, definitely” answer. Finally, we created the binary variable *Any Negative Experience*, which indicated if the response to a PREM item was other than “yes, definitely.”

### Background Variables

We recorded respondents’ sociodemographic variables, such as age, gender (female or male), education (primary, secondary, or tertiary), family status (married or not married), employment status (with a paid job or without a paid job including students, pensioners, unemployed, etc), and place of residence (capital, other cities, or village). Age groups were formed according to main Medical Subject Heading (MeSH) categories, adding 18-year olds to the young adult category (young adults: 18–24-year olds; adults: 25–44-year olds; middle aged: 45–64-year-olds; aged > 80: 65+ year-olds) [46]. Net monthly household income was queried in 11 range categories, and per-capita household income was calculated by dividing the category midrange values by the number of household members, without adjustment for the number of children. The midrange value of the upper open category was calculated by fitting the Pareto curve as proposed by Parker and Fenwick [47]. We generated income groups according to quintiles of per-capita monthly net household income, with lower limits of €203 (US $241), €285 (US $338), €365 (US $432), and €463 (US $549), respectively, for the second, third, fourth, and fifth quintiles calculated from the third to eighth national decile group means by linear interpolation [48]. We also recorded respondents’ health status using the Minimal European Health Module (MEHM) [49,50]. The MEHM included an item on self-perceived health (very bad, bad, fair, good, or very good); an item on whether the respondent had long-standing health problems (1=chronic morbidity present); and the Global Activity Limitation Indicator, which assessed for activity limitations due to health problems (not limited at all, limited but not severely, or severely limited) [50].

### Statistical Methods

Descriptive methods were used when analyzing the sociodemographic characteristics of the sample as well as the PREM items. To test the basic psychometric properties of the PREM scores constructed from multiple items, we assessed their distributional properties, performed exploratory factor analysis (EFA), and calculated internal consistency (Cronbach α) [51]. The normality assumption was tested via the Shapiro–Wilk test [52] and the Kaiser–Meyer–Olkin (KMO) test was used to check the suitability of data for EFA [53]. Pairwise biserial or polychoric correlations were calculated between PREM items and eHEALS scores. Polychoric correlation assumes bivariate normally distributed latent variables behind ordinal response items and provides the correlation coefficients for those latent variables using a maximum-likelihood estimation [54]. The bivariate associations between eHEALS quartiles and PREM scores as well as demographic variables were tested via ANOVA, the Kruskal–Wallis test, and the chi-square test of independence [55-57].

We performed multivariate regression analyses to explore the relationship between eHEALS quartiles and PREM items, as well as the composite PREM scores, after controlling for sociodemographic variables, respondents’ health status (MEHM), the setting of the visit (GP, public specialist, or private specialist), and type of HCP (GP, specialist, or other allied health professional). The following models were conducted: (1) logistic regression for binary PREM items or constructed binary variables, (2) ordered logit models for polytomous PREM items, and (3) ordinary least squares (OLS) models for waiting times and composite PREM scores. We tested the joint significance of eHEALS quartiles as a single predictor variable using the Wald test. OLS models were tested for heteroskedasticity via the Breusch–Pagan test and for specification error via the Ramsey regression equation specification error test (RESET). We applied robust regression if heteroskedasticity was detected [58]. In case we detected model functional misspecification error, log-transformation or square-root transformation was performed on the dependent variable [59]. Goodness of fit of logistic and ordered logit models were tested, respectively, by the binary and ordinal versions of the Hosmer–Lemeshow test [60]. Unmet medical needs were also explored in an extended sample of those respondents who experienced health problems over the past 12 months, regardless of whether they had ambulatory consultation with an HCP. All calculations were performed using the Stata version 14.2 statistical software package (StataCorp) [61]. The level of significance was set at *P*<.05, and we applied no more than 15 observations per predictor variable when running multiple regression models [62]. Analyses were carried out without applying weights on the sample.

## Results

### Respondents’ Characteristics

From the 1000 survey respondents, 736 had ambulatory HCP consultation within 12 months, out of which 5 happened over telephone. In 118 cases the respondent did not have a health problem, and 25 respondents could not tell if the visit happened at the regular HCP. After applying all criteria in sequence, 666 individuals were included in the sample (Table 1). Respondents with tertiary education and from the highest income quintile were slightly over-represented, whereas rural citizens were slightly under-represented compared with the general population. Mean age of our sample was 48.9 (SD 17.6) years. The demographic characteristics of the sample, all survey respondents, and the general population are summarized in Table 1. Responses on the income, chronic morbidity, and activity limitation items were provided by 86.5% (576/666), 89.0% (593/666), and 96.4% (642/666) of the respondents, respectively. The first, second, third, fourth, and fifth income quintiles of the sample corresponded to €115 (US $136), €247 (US $293), €332 (US $393), €397 (US $470), and €669 (US $786) mean per-capita household income levels, using the April 2020 12-month-average exchange rate of 330.73 HUF/€ (279.14 HUF/US $) [63]. The responses by PREM items are summarized in Table 2.

**Table 1:** Sample characteristics.

Characteristics				Sample (N=666), n (%)		Survey (N=1000), n (%)		General adult population [42], %
**Sociodemographic**								
	**Age group**							
		18-24	62 (9.3)		118 (11.8)		10.6	
		25-44	234 (35.1)		389 (38.9)		35.7	
		45-64	191 (28.7)		272 (27.2)		33.1	
		65+	179 (26.9)		221 (22.1)		20.6	
	**Gender**							
		Female	364 (54.7)		550 (55.0)		53.4	
		Male	302 (45.4)		450 (45.0)		46.6	
	**Education**							
		No primary school	—^a^		—		0.6	
		Primary	213 (31.9)		341 (34.1)		48.1	
		Secondary	244 (36.6)		363 (36.3)		33.5	
		Tertiary	209 (31.4)		296 (29.6)		17.8	
	**Household income per capita**							
		First quintile	142 (21.3)		228 (22.8)		20.0	
		Second quintile	105 (15.8)		167 (16.7)		20.0	
		Third quintile	57 (8.6)		81 (8.1)		20.0	
		Fourth quintile	86 (12.9)		118 (11.8)		20.0	
		Fifth quintile	186 (27.9)		254 (25.4)		20.0	
		Missing^b^	90 (13.5)		152 (15.2)		—	
	**Family status**							
		Married/domestic partnership	432 (64.9)		618 (61.8)		—	
		Single/divorced/widow	234 (35.1)		382 (38.2)		—	
	**Employment status**							
		Paid job	319 (47.9)		500 (50.0)		48.3	
		Without paid job	347 (52.1)		500 (50.0)		51.7	
	**Residence**							
		Budapest	146 (21.9)		213 (21.3)		17.4	
		City	371 (55.7)		557 (55.7)		52.1	
		Village	149 (22.4)		230 (23.0)		30.5	
	**NUTS^c^ 1 region**							
		Central Hungary	236 (35.4)		348 (34.8)		30.0	
		Transdanubia	237 (35.6)		299 (29.9)		30.4	
		Great Plain and North	193 (28.9)		353 (35.3)		39.6	
**MEHM^d^**								
	**Self-perceived health**							
		Very bad	3 (0.5)		5 (0.5)		—	
		Bad	62 (9.3)		77 (7.7)		—	
		Fair	252 (37.8)		323 (32.3)		—	
		Good	293 (43.9)		471 (47.1)		—	
		Very good	56 (8.4)		124 (12.4)		—	
	**Chronic morbidity**							
		No	200 (30.0)		390 (39.0)		—	
		Yes	393 (59.0)		489 (48.9)		—	
		Missing	73 (10.9)		121 (12.1)		—	
	**Activity limitations**							
		Not limited at all	342 (51.4)		579 (57.9)		—	
		Limited but not severely	254 (38.1)		313 (31.3)		—	
		Severely limited	46 (6.9)		56 (5.6)		—	
		Missing	24 (3.6)		52 (5.2)		—	
**Inclusion criteria**								
	**Ambulatory HCP^e^ visit in past 12 months**							
		No/not face-to-face/missing	0 (0.0)		269 (26.9)		—	
		Yes, but not for own health problem	0 (0.0)		52 (5.2)		—	
		Yes, at regular HCP	546 (81.9)		546 (54.6)		—	
		Yes, but not at regular HCP	120 (18.0)		120 (12.0)		—	
		Yes, missing if regular HCP	0 (0.0)		13 (1.3)		—	

^a^Not available.

^b^Missing: missing responses/do not know/do not want to answer.

^c^NUTS: Nomenclature of Territorial Units for Statistics.

^d^MEHM: Minimal European Health Module.

^e^HCP: health care professional.

**Table 2:** Patient responses by PREM^a^ items (N=666).

Patient response			n (%)
**Access to care: last visit**			
	**Health care setting**		
		GP^b^	278 (41.7)
		Public specialist	316 (47.4)
		Private specialist	72 (10.8)
	**Type of HCP^c^**		
		GP	278 (41.7)
		Specialist	360 (54.1)
		Nurse/other HCP	28 (4.2)
	**Time of last visit**		
		In the last 30 days	277 (41.6)
		Between 1 and 3 months ago	180 (27.0)
		Between 3 and 6 months ago	95 (14.3)
		Between 6 and 12 months ago	114 (17.1)
**Access to care: unmet medical needs**			
	**Missed visit due to travel burden**		
		No	506 (76.0)
		Yes	147 (22.1)
		Missing^d^	13 (2.0)
	**Missed visit due to cost burden**		
		No	534 (80.2)
		Yes	120 (18.0)
		Missing	12 (1.8)
	**Missed intervention due to cost burden**		
		No	559 (83.9)
		Yes	99 (14.9)
		Missing	8 (1.2)
	**Missed medication due to cost burden**		
		No	508 (76.3)
		Yes	148 (22.2)
		Missing	10 (1.5)
**Access to care: waiting times**			
	**Problem with waiting to be seen on the day of consultation**		
		No	487 (73.1)
		Yes	179 (26.9)
	**Problem with waiting for appointment**		
		No	564 (84.7)
		Yes	102 (15.3)
**Patient experiences**			
	**Doctor spending enough time with patient in consultation**		
		Yes, definitely	427 (64.1)
		Yes, to some extent	160 (24.0)
		No, not really	57 (8.6)
		Definitely not	17 (2.6)
		Missing	5 (0.8)
	**Doctor providing easy to understand explanations**		
		Yes, definitely	459 (68.9)
		Yes, to some extent	166 (24.9)
		No, not really	27 (4.1)
		Definitely not	12 (1.8)
		Missing	2 (0.3)
	**Doctor giving opportunity to ask questions or raise concerns**		
		Yes, definitely	414 (62.2)
		Yes, to some extent	164 (24.6)
		No, not really	63 (9.5)
		Definitely not	15 (2.3)
		Missing	10 (1.5)
	**Doctor involving patient in decisions about care and treatment**		
		Yes, definitely	338 (50.8)
		Yes, to some extent	195 (29.3)
		No, not really	77 (11.6)
		Definitely not	19 (2.9)
		Missing	37 (5.6)
	**Overall quality of the visit**		
		Poor	19 (2.9)
		Fair	60 (9.0)
		Good	186 (27.9)
		Very good	205 (30.8)
		Excellent	193 (29.0)
		Missing	3 (0.5)

^a^PREM: OECD-proposed set of questions on Patients’ Experiences with Ambulatory Care.

^b^GP: general practitioner.

^c^HCP: health care professional.

^d^Missing: missing responses/do not know/do not want to answer.

### eHEALS

Mean eHEALS score was 29.3 (SD 4.9). eHEALS quartile mean scores were as follows: first quartile 23.5 (range 12-26; 191/666, 28.7%), second quartile 28.2 (range 27-29; 151/666, 22.7%), third quartile 31.2 (range 30-32; 182/666, 27.3%), and fourth quartile 36.0 (range 33-40; 142/666, 21.3%). Mean age of individuals in the fourth eHEALS quartile was 44.5 years (SD 17.1), which was lower than that of individuals in the first (49.9 years [SD 17.4]), second (51.2 [SD 17.5]), and third quartiles (49.3 [SD 17.7; *F*_3,662_=4.07, *P*=.007). Mean eHEALS scores did not differ between male and female respondents (*t*_664_=1.27, *P*=.21). However, while the percentage of female respondents decreased evenly from the first (101/364, 27.8%), second (92/356, 25.8%), third (86/364, 23.6%), and fourth eHEALS quartiles (85/364, 23.4%), male respondents were concentrated in the first (90/302, 29.8%) and third (96/302, 31.8%) quartiles (χ^2^_3_=8.2, *P*=.04). The difference in terms of education (χ^2^_6_=5.6, *P*=.47) and income (χ^2^_12_=7.9.6, *P*=.79) was not significant between the four eHEALS groups.

### PREM Unmet Medical Needs

A majority of respondents (380/631, 60.2%) did not report unmet medical needs in any areas. One unmet need was reported by 18.5% (117/631), 2 unmet needs by 8.9% (56/631), 3 unmet needs by 7.4% (47/631), and 4 unmet needs by 4.9% (31/631) of respondents. The Unmet Medical Needs Score had a single-factor structure with a KMO value of 0.73, suggesting moderately adequate sampling for EFA. The Cronbach α of .73 suggested acceptable internal consistency of this score constructed by adding the PREM items of the Unmet Medical Needs section.

### PREM Waiting Times

Mean office waiting times were 63.3 (SD 71.0) minutes; 23.0% (152/661) of respondents waited less than 15 minutes, while waiting time was longer than 2 hours for 14.2% (94/661) of the sample. Long office waiting time was a problem for 26.9% (179/666) of all respondents, and for 34.8% (179/514) of those who waited longer than 15 minutes. Mean appointment waiting time was 16.8 (SD 27.8) days. Whereas 37.6% (242/643) of the sample could get an appointment on the same day, 18.2% (117/643) of respondents had to wait longer than 30 days. Long appointment waiting time was a problem for 15.3% (102/666) of all respondents, and for 24.1% (102/424) of those who did not get appointment on the same day. Any waiting problem either at the HCP office or before getting an appointment was reported by 33.5% (223/666) of the sample.

### PREM Patient Experiences

The Problem Score showed strong right skew (mean 2.0, SD 2.5; median 1; kurtosis 4.5; skewness 1.4; Shapiro–Wilk test *P*<.001). EFA suggested a single-factor structure with adequate sampling (KMO statistic=0.82) and good internal consistency (Cronbach α=.87). Whereas 0.5% (3/623) of respondents indicated the worst experience in all domains (*definitely not* answers for all 4 items; score 16), the experience was flawless (*definitely yes* answers for all 4 items; score 4) for 40.9% (255/623). The Negative Experiences Score had bimodal distribution (mean 1.5, SD 1.6; kurtosis 1.7; skewness 0.5; Shapiro–Wilk test *P*<.001) and a single-factor structure with adequate sampling (KMO statistic=.80) and good internal consistency (Cronbach α=.83). Problems were reported in 0, 1, 2, 3, and 4 domains by 40.9% (255/623), 18.0% (112/623), 11.2% (70/623), 11.4% (71/623), and 18.5% (115/623) of respondents, respectively. The strong correlation between the Problem Score and Negative Experiences Score (polyserial ρ=0.95) suggested that counting the domains with answers other than *definitely yes* accounted for most of the information within the patient experiences section.

### Correlation Between PREM and eHEALS Scores

The polychoric correlation matrix of PREM items and eHEALS score is shown in Table 3. The patient experience measures were strongly intercorrelated, whereas the correlation between those PREMs and waiting times and unmet medical need measures were moderate or weak. The corresponding waiting times and waiting problems were strongly correlated. The overall quality of the visit showed a strong negative correlation with items of the patient experiences section of the survey; the correlation was moderate or weak with remaining items. The eHEALS score showed a weak negative correlation with any of the PREMs. The correlation between PREM items and the time of the last visit was minimal.

**Table 3:** Correlation matrix of PREM^a^ items.^b^

Variable	Patient experiences					Access to care							
						Waiting times				Unmet medical needs			
	Time	Understand	Questions	Decisions	Overall quality	oWT	oWP	aWT	aWP	Travel	Visit	Intervention	Medication
Time^c^	1.00												
Understand^d^	0.75	1.00											
Questions^e^	0.77	0.77	1.00										
Decisions^f^	0.71	0.76	0.83	1.00									
Overall quality^g^	–0.79	–0.75	–0.78	–0.74	1.00								
oWT^h^	0.35	0.30	0.34	0.24	–0.36	1.00							
oWP^i^	0.42	0.43	0.38	0.38	–0.45	0.67	1.00						
aWT^j^	0.17	0.16	0.11	0.14	–0.12	0.11	0.15	1.00					
aWP^k^	0.42	0.32	0.38	0.35	–0.37	0.32	0.50	0.67	1.00				
Travel^l^	0.20	0.21	0.21	0.24	–0.16	0.17	0.33	0.05	0.38	1.00			
Visit^m^	0.36	0.30	0.36	0.27	–0.32	0.14	0.40	0.10	0.36	0.61	1.00		
Intervention^n^	0.17	0.16	0.20	0.19	–0.18	0.12	0.37	0.18	0.44	0.61	0.89	1.00	
Medication^o^	0.22	0.23	0.26	0.16	–0.17	0.16	0.33	0.15	0.43	0.47	0.66	0.67	1.00
eHEALS^p^	–0.03	–0.13	–0.04	–0.04	0.11	0.01	0.14	–0.04	–0.02	–0.02	0.00	0.02	–0.06
Last visit^q^	0.03	-0.01	0.00	0.01	–0.06	0.00	0.06	–0.10	–0.08	0.00	–0.07	–0.10	–0.04

^a^PREM: OECD (Organisation for Economic Co-operation and Development)-proposed set of questions on Patients’ Experiences with Ambulatory Care.

^b^Pairwise tetrachoric correlations for binary item pairs, polychoric correlations for polytomous items, polyserial and biserial correlations between eHEALS scores and polytomous and binary items, respectively.

^c^Doctor spending enough time with patient in consultation (4-point Likert scale; higher points indicate more problems).

^d^Doctor providing easy-to-understand explanations (4-point Likert scale; higher points indicate more problems).

^e^Doctor giving opportunity to ask questions or raise concerns (4-point Likert scale; higher points indicate more problems).

^f^Doctor involving patient in decisions about care and treatment (4-point Likert scale; higher points indicate more problems).

^g^Overall quality of last appointment (5-point Likert scale; higher points indicate better experience).

^h^Waiting time to be seen on the day of consultation (office waiting time [oWT]).

^i^Problem with waiting to be seen on the day of consultation (office waiting was a problem [oWP]).

^j^Waiting time to get the appointment (appointment waiting time [aWT]).

^k^Problem with waiting for appointment: yes (appointment waiting time was a problem [aWP]).

^l^Missed visit due to travel burden.

^m^Missed visit due to cost burden.

^n^Missed intervention due to cost burden.

^o^Missed medication due to cost burden.

^p^eHEALS: eHealth Literacy Scale.

^q^Time of last visit: 4 categories; higher points indicate more time elapsed since last visit.

### Bivariate Association Between PREM and eHEALS Quartiles

**Figure 1:**
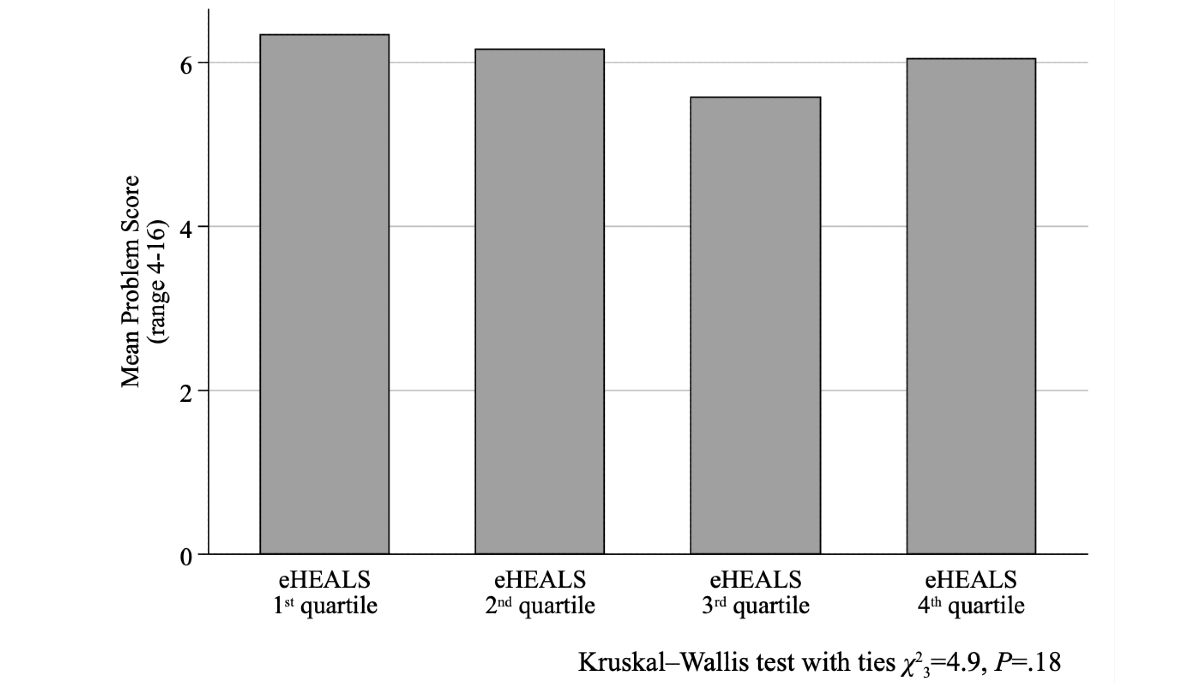
Problem score by eHEALS (eHealth Literacy Scale) quartiles.

**Figure 2:**
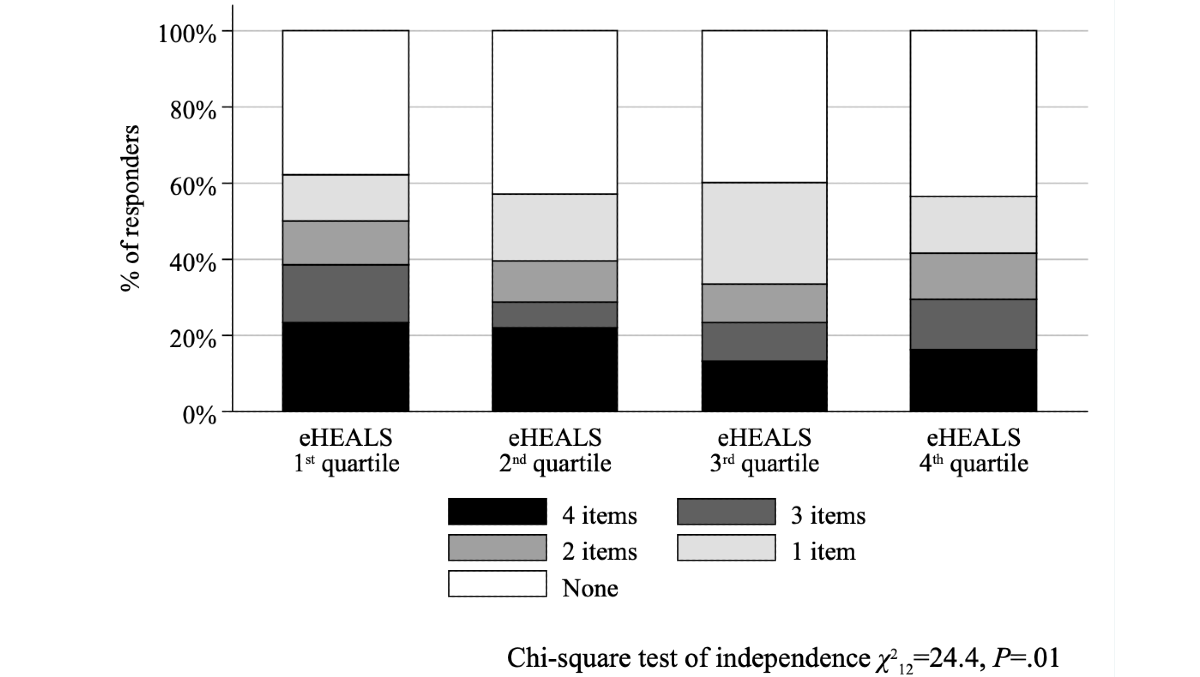
Negative Experiences Score by eHEALS (eHealth Literacy Scale) quartiles.

**Figure 3:**
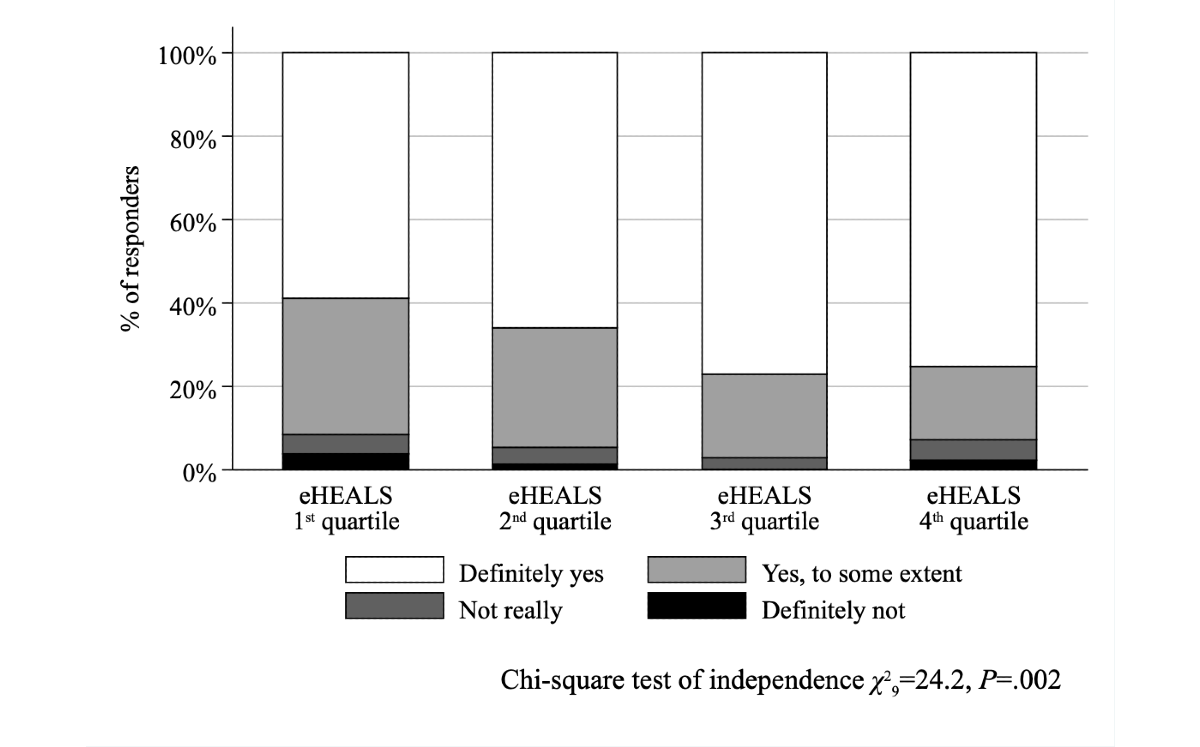
Perceived easiness of understanding the explanations of the health care professional by eHEALS (eHealth Literacy Scale) quartiles.

**Figure 4:**
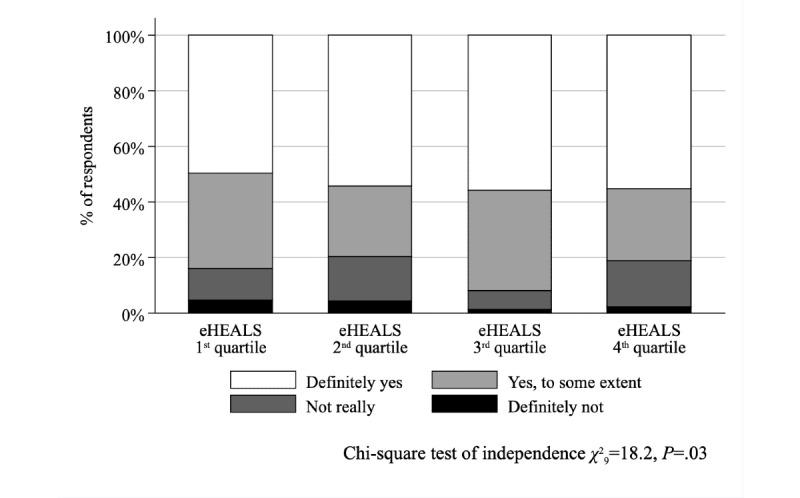
Perceived involvement of the respondent by the health care professional in decisions about care and treatment by eHEALS (eHealth Literacy Scale) quartiles.

**Figure 5:**
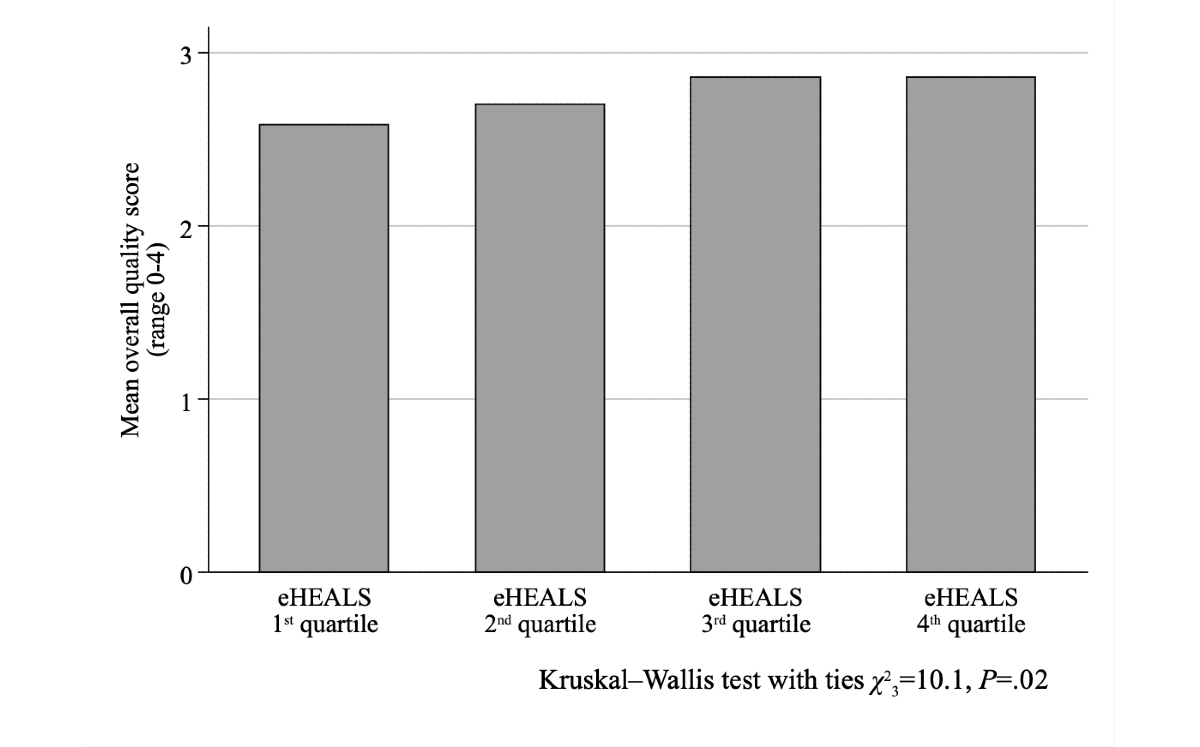
Mean overall quality of the last visit by eHEALS (eHealth Literacy Scale) quartiles.

**Figure 6:**
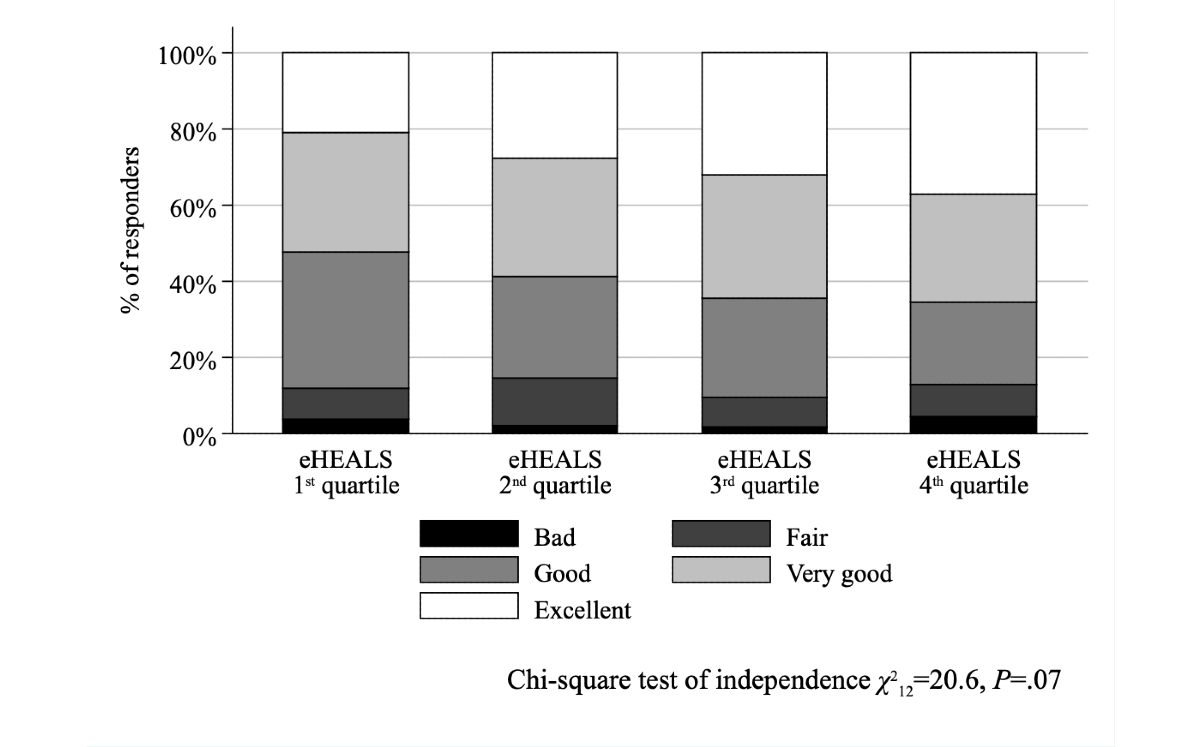
Overall quality categories of the last visit by eHEALS (eHealth Literacy Scale) quartiles.

The association between eHEALS quartiles and the problem score was not significant (Kruskal–Wallis test with ties, χ^2^_3_=4.9, *P*=.18; Figure 1); conversely, the association was significant (χ^2^_12_=24.4, *P*=.01) when the negative experiences score was considered (Figure 2). While we found no relationship between eHEALS quartiles and the time spent with the patient (χ^2^_9_=14.5, *P*=.11) and opportunity to ask questions (χ^2^_9_=9.2, *P=*.42), the association was significant (χ^2^_9_=24.2, *P*=.002) between how easy it was to understand the HCP’s explanations (Figure 3) and the extent to which the HCP involved the respondent in decisions (χ^2^_9_=18.2, *P*=.03; Figure 4). The association between eHEALS quartiles and the overall quality score was significant (Kruskal–Wallis test with ties χ^2^_3_=10.1, *P*=.02; Figure 5). By contrast, the association between eHEALS quartiles and the share of respondents by overall quality categories was not significant (χ^2^_12_=20.6, *P*=.07; Figure 6). Although the differences were small, the results suggest that respondents in the lowest eHEALS quartile had the least positive experience with HCP communication. Positive experiences were most frequently reported in the third and fourth eHEALS quartiles, whereas the subgroup with the highest eHEALS scores reported somewhat more negative experiences, when compared with respondents in the third eHEALS quartile. We found no association between eHEALS quartiles and either unmet medical needs or waiting times.

### Regression Analyses of Individual PREM Items

After controlling for sociodemographic variables, respondents’ health status, the setting of the visit, and type of HCP in ordered logit models (Table 4), the association was significant between eHEALS quartiles and the “Easy to understand explanations” item (Wald test χ^2^_3_=11.8, *P*=.008). Although eHEALS quartiles jointly were not significant predictors of the “Opportunity to ask questions” item (Wald test χ^2^_3_=4.7, *P*=.19), the differences between the first and third eHEALS quartiles were significant in both of those items (*P*<.001 and *P*=.04, respectively). The association was not significant between eHEALS quartiles and either the time spent with the patient (Wald test χ^2^_3_=1.8, *P*=.61) or the involvement of the patient in decisions about care and treatment (Wald test χ^2^_3_=3.4, *P*=.33).

After controlling for covariates, the overall quality also differed between respondents in the first and third eHEALS quartiles (Table 5), although the joint effect of eHEALS quartiles on the overall quality was not significant (Wald test χ^2^_3_=6.0, *P*=.11). Unmet medical needs (Multimedia Appendix 3) and waiting times (Multimedia Appendix 4) were not associated with the eHEALS quartiles. The sensitivity analysis has shown similar findings in the majority of models using alternative eHEALS group boundaries (Multimedia Appendix 6). In half of the alternative scenarios the probability of missed interventions was also significantly lower (*P*=.05 to *P*=.02) in the third (moderately high) than in the first (lowest) and eHEALS score group, but not in the predefined quartiles (*P*=.05). We did not find significant (*P*=.16 to *P*=.88) association between eHEALS quartiles and any unmet needs variables in the extended sample involving those respondents who had a health problem over the past 12 months, regardless their participation in ambulatory HCP consultation (Multimedia Appendix 7). The detailed analysis of the effect of covariates on the PREM modules was out of scope for this paper, and has been provided elsewhere [30,41,64].

**Table 4:** Ordered logit regression of the patient experience PREM^a^ items.

Variables		Time^b^			Understand^c^			Questions^d^			Decisions^e^	
		β	*P* value	β		*P* value	β		*P* value	β		*P* value
**eHealth Literacy Scale^f^**												
	Second quartile	–0.30	.28	–0.29		.31	–0.42		.12	–0.20		.47
	Third quartile	–0.31	.24	–0.98		<.001	–0.54		.04	–0.30		.23
	Fourth quartile	–0.14	.61	–0.51		.09	–0.26		.34	0.15		.57
**Age group^g^**												
	25-44 years old	–0.87	.02	–0.78		.04	–0.81		.03	–0.73		.05
	45-64 years old	–1.06	.009	–1.45		<.001	–1.38		<.001	–0.88		.03
	65+ years old	–1.38	.002	–1.81		<.001	–1.58		<.001	–1.42		<.001
**Education^h^**												
	Secondary	–0.27	.31	0.10		.71	–0.03		.90	–0.01		.98
	Tertiary	0.25	.37	0.22		.47	0.14		.62	0.08		.78
**Gender**												
	Male	–0.37	.09	0.12		.59	–0.20		.36	–0.08		.68
**Income^i^**												
	Second quintile	–0.01	.97	–0.31		.36	0.32		.31	0.40		.19
	Third quintile	0.22	.57	–0.17		.67	0.50		.17	0.53		.14
	Fourth quintile	0.25	.47	–0.22		.54	0.24		.49	0.38		.25
	Fifth quintile	0.08	.8	0.07		.83	0.30		.32	0.49		.10
**Paid employment**												
	Yes	–0.34	.19	–0.14		.60	–0.13		.59	–0.14		.58
**Family status**												
	Married/domestic partnership	–0.27	.21	–0.22		.32	0.01		.97	–0.12		.55
**Residence^j^**												
	City	–0.07	.76	–0.20		.44	0.10		.67	0.21		.37
	Village	–0.70	.03	–0.34		.31	–0.29		.34	–0.59		.06
**Self-perceived health^k^**												
	Very bad	–13.30	.99	–12.77		.99	0.13		.92	0.03		.98
	Bad	1.20	.03	1.67		.02	0.72		.19	1.46		.01
	Fair	0.52	.26	1.80		.003	0.41		.35	0.96		.04
	Good	0.35	.41	1.35		.02	0.15		.71	0.63		.14
**Global Activity Limitation Indicator^l^**												
	Limited but not severely	0.26	.27	0.15		.54	0.11		.62	0.22		.34
	Severely limited	0.19	.66	0.12		.80	0.33		.44	0.23		.56
**Chronic morbidity**												
	Yes	0.18	.49	0.20		.48	0.29		.27	0.39		.12
**Setting^m^**												
	Public specialist	1.23	.009	1.23		.01	—^n^		.99	0.37		.46
	Private specialist	0.90	.11	1.00		.08	–0.37		.53	0.34		.54
**HCP type^o,p^**												
	Specialist	–1.39	.003	–1.32		.005	–0.41		.42	–0.83		.09
	Nurse/other HCP	—	—	—		—	—		—	—		—
**Regular HCP**												
	Yes	–0.29	.28	0.05		.87	–0.35		.18	–0.27		.31
N		502		504			500			477		
LR^q^ test *χ*^2^_28_		52.7	.003	60.6		<.001	43.7		.03	50.7		.005
GOF^r^ test *χ*^2^_26_		18.5	.86	13.5		.98	21.7		.71	24.9		.52

^a^PREM: OECD (Organisation for Economic Co-operation and Development)-proposed set of questions on Patients’ Experiences with Ambulatory Care.

^b^Doctor spending enough time with patient in consultation (4-point Likert scale).

^c^Doctor providing easy to understand explanations (4-point Likert scale).

^d^Doctor giving opportunity to ask questions or raise concerns (4-point Likert scale).

^e^Doctor involving patient in decisions about care and treatment (4-point Likert scale).

^f^Base: first quartile.

^g^Base: 18-24 years old.

^h^Base: primary.

^i^Base: first quintile.

^j^Base: capital.

^k^Base: very good.

^l^Base: not limited.

^m^Base: general practitioner.

^n^Not available.

^o^Base: general practitioner.

^p^HCP: health care professional.

^q^Likelihood ratio; omnibus test for independence, current model versus null model.

^r^Goodness of fit; ordinal version of the Hosmer–Lemeshow test.

**Table 5:** Multivariate regression of PREM^a^ scores.

Model		Overall quality		Log-problem score		Negative experience score			Any negative experience		
		Ordered logit		Robust^b^		Robust			Logistic		
		β	*P* value	β	*P* value		β	*P* value		β	*P* value
**eHEALS^c,d^**											
	Second quartile	0.24	.31	–0.06	.23		–0.37	.08		–0.25	.38
	Third quartile	0.55	.02	–0.10	.02		–0.46	.02		–0.16	.54
	Fourth quartile	0.34	.16	–0.02	.74		–0.17	.40		–0.17	.56
**Age group^e^**											
	25–44 years old	0.56	.09	–0.15	.03		–0.46	.08		–0.64	.14
	45–64 years old	0.71	.04	–0.22	.002		–0.83	.003		–1.15	.01
	65+ years old	1.12	.003	–0.29	<.001		–1.16	<.001		–1.60	.001
**Education^f^**											
	Secondary	–0.12	.60	—^g^	.97		–0.01	.96		–0.04	.89
	Tertiary	–0.39	.10	0.04	.36		0.18	.37		–0.03	.92
**Gender**											
	Male	0.07	.69	–0.03	.42		–0.03	.86		0.21	.32
**Income^h^**											
	Second quintile	–0.10	.70	0.04	.35		0.22	.29		0.71	.03
	Third quintile	0.17	.59	0.05	.40		0.24	.39		0.78	.04
	Fourth quintile	–0.01	.97	0.02	.69		0.20	.41		0.44	.20
	Fifth quintile	0.06	.81	0.05	.33		0.27	.21		0.61	.047
**Paid employment**											
	Yes	0.12	.59	–0.04	.31		–0.08	.64		0.02	.93
**Family status**											
	Married/domestic partnership	0.25	.17	–0.03	.45		–0.09	.55		0.12	.59
**Residence^i^**											
	City	–0.04	.85	—	.99		0.03	.85		0.10	.70
	Village	0.26	.34	–0.11	.03		–0.49	.02		–0.66	.03
**Self-perceived health^j^**											
	Very bad	0.21	.88	–0.05	.72		0.04	.95		0.01	.99
	Bad	–0.97	.047	0.24	.007		1.24	<.001		1.32	.02
	Fair	–1.00	.01	0.15	.02		0.80	.003		0.69	.11
	Good	–0.85	.02	0.10	.09		0.59	.01		0.49	.21
**Activity limitations^k^**											
	Limited but not severely	–0.27	.18	0.04	.28		0.17	.34		0.34	.14
	Severely limited	–0.23	.55	0.05	.48		0.20	.52		–0.08	.85
**Chronic morbidity**											
	Yes	0.04	.85	0.04	.32		0.15	.41		0.03	.91
**Setting^l^**											
	Public specialist	–0.60	.16	0.18	.09		0.49	.22		0.95	.12
	Private specialist	–0.05	.91	0.13	.24		0.22	.62		0.29	.66
**HCP type^m,n^**											
	Specialist	0.72	.09	–0.24	.02		–0.75	.05		–1.19	.046
	Nurse/other HCP	—	—	—	—		—	—		—	—
**Regular HCP**											
	Yes	0.03	.91	–0.05	.24		–0.25	.22		–0.36	.21
Constant		—	—	1.87	<.001		1.82	<.001		0.81	.25
N		503		473			473			505	
LR^o^ test *χ*^2^_28_		42.1	.04							49.6	.007
LR test *F*_28,444_				2.63	<.001		2.27	<.001			
R^2^				0.13			0.13				
GOF^p^ test *χ*^2^_35_		30.5	.68								
GOF test *χ*^2^_470_										503.3	.14
Ramsey RESET^q^ *F*_3,434_				2.37	.07		0.07	.98			

^a^PREM: OECD-proposed set of questions on Patients’ Experiences with Ambulatory Care.

^b^Ordinary least squares (OLS) regression with robust standard errors.

^c^Base: first quartile.

^d^eHEALS: eHealth Literacy Scale.

^e^Base: 18-24 years old.

^f^Base: Primary.

^g^Not available.

^h^Base: first quintile.

^i^Base: Capital.

^j^Base: Very good.

^k^Base: Not limited.

^l^Base: General practitioner.

^m^Base: General practitioner.

^n^HCP: health care professional.

^o^Likelihood ratio; omnibus test for independence, current model versus null model.

^p^Goodness of fit; Hosmer–Lemeshow test.

^q^Regression equation specification error test.

### Regression Analyses of Composite PREM Scores

The specification of robust linear regression models was acceptable for the log-problem score and the negative experience score (Table 5). Findings show that the difference was significant between the first and third eHEALS quartiles in the log-problem score (*P*=.02) and negative experience score models (*P*=.02). The joint Wald test of eHEALS quartiles was not significant in either model (log-problem score *F*_3,430_=2.28, *P*=.08; negative experience score *F*_3,430_=2.17, *P*=.09; any negative experience χ^2^_3_=0.8, *P*=.84). In addition, logistic regression models for *any unmet medical needs* and *any waiting problems* had an acceptable fit (Multimedia Appendix 5); eHEALS was not a significant predictor in any of these models (*P*=.05 to *P*=.42). In several scenarios of the sensitivity analysis, the *unmet medical needs score* and *any unmet medical needs* suggested less unmet needs in the third (moderately high) than in the first (lowest) eHEALS score groups (Multimedia Appendix 6).

## Discussion

To our knowledge, this is the first study that explores the relationship between eHealth literacy and PREMs with outpatient care. Our findings show a weak concave relationship between eHEALS scores and PREMs. We observed significant differences between respondents with lowest self-reported eHealth literacy levels (first eHEALS quartile) and the ones with moderately high levels (third eHEALS quartile) in terms of how easy it was to understand the explanations of the HCP, having the opportunity to ask questions, the number of items where respondents experienced problems, and the overall quality of the last visit. Sensitivity analysis using alternative boundaries between eHEALS groups confirmed these findings in multiple alternative scenarios. Although the bivariate association between eHealth literacy and the involvement of respondents in decision making was significant, after controlling for covariates in multiple regression analyses, respondents’ perception of spending enough time in the consultation and involvement in decision making did not show a statistically significant relationship with the eHEALS scores. Besides, our findings show no significant association between eHealth literacy and unmet medical needs and waiting times.

Although our literature search did not reveal papers reporting the association between PREM and eHealth literacy, several studies explored the effect of eHealth literacy (measured with the eHEALS instrument) on aspects of people-centered care such as the patient–physician relationship. A study among Iranian patients with multiple myeloma found a positive relationship between eHealth literacy and shared decision making, where eHealth literacy had a direct positive influence on shared decision making and an indirect positive effect mediated by collaborative patient communication patterns and trust in the health care system [37]. In a large survey among the Israeli general population, higher eHealth literacy score was associated with a more extensive interaction and a more balanced power position vis-à-vis with the treating physician [22]. While eHealth literacy had a direct positive influence on productive functional behaviors in all domains of patient empowerment among members of a Slovenian online health community, it also had a moderating effect on both dysfunctional and functional behaviors [65]. Using the Health Literacy Questionnaire, a survey on a large Dutch online panel of health care users demonstrated positive relationship between information appraisal, a higher-order health literacy skill, and shared decision making [66,67].

Our results show a strong negative correlation between the overall quality of the visit and the perceived problems with HCP communication including the involvement of the respondents in decision making. However, the relationship between overall patient-reported experience and eHealth literacy was not linear. The slightly increased probability of negative patient experiences among respondents with highest eHEALS scores is in line with the findings of a large international qualitative study among online health information users, where participants frequently reported reluctance to discuss the online content due to the expected negative reception from their HCPs [68]. On the same note, a systematic review on the impact of online health information on patient–physician relationship identified a positive effect on the majority of the cases, although several studies reported negative feelings concerning the discussion of online information with HCPs [33]. In the 2007 Health Information National Trends Survey, patients’ concerns about the quality of online health information increased the likelihood of discussing it with their HCPs, while they were also more likely to experience negative reactions from the HCPs concerning the shared information [36].

Recognizing the multidimensional determinants of the patient–physician interaction, a recent line of research aimed to establish patient profiles characterized by various skill levels and attitudes, including eHealth literacy [32,65,69]. In a large multicountry survey, 4 distinct patient decision styles were described. While patients with a passive decision-making style had the lowest eHealth literacy skills, the autonomous-collaborator group showed somewhat higher eHealth literacy and worse patient–physician communication, compared with that of the collaborators, who were most likely to engage in shared decision making [32].

Among several potential contributing factors, the emergence of negative experiences among patients with greater eHealth literacy levels may partly be explained by the properties of the eHEALS instrument. Showing low correlation with objective measures of eHealth literacy, eHEALS has been described as a tool measuring rather self-efficacy related to eHealth literacy than actual skills [70]. Patients with low functional health literacy presenting high eHEALS scores tended to rely on non-established criteria when evaluating online health information compared to ones with high functional health literacy, who relied on more established criteria [71]. Overconfident use of low-quality health information due to ignorance about the actual low skill level [72] combined with high psychological empowerment may lead to dangerous self-management [73], evoking negative reactions from HCPs. Therefore, since the original definition coined by Norman and Skinner in 2006 [14], efficient communication skills or a supportive patient–HCP relationship has been included in several updated concepts of eHealth literacy [74] and eHealth readiness [69].

We also assume that access to high-quality online information including international best-in-class services may raise the expectations of people that may contrast their real-world experiences with the Hungarian health system, which operates at a lower efficiency and expenditure levels compared with other high-income societies [7]. Besides, GP gatekeeping systems, such as the Hungarian one, have been designed to restrict the demand side of health care, and are perceived as being less patient centric than non-gatekeeping systems [29]. It has been shown that patients that face barriers to access to care are usually more prone to health information–seeking behaviors [75]. Furthermore, dissatisfaction with the patient-centeredness of physicians and high eHealth literacy were among the key reasons of postvisit online information seeking in a US online health community [76].

Although the relationship between eHealth literacy and unmet medical needs or waiting times was not significant in our sample, we found the highest eHEALS scores among respondents with worst self-reported health [39]. It has been demonstrated that chronic patients develop health literacy skills over time [77], and higher health literacy was associated with better outcomes even in difficult-to-treat patients [78]. By contrast, multimorbid patients often experience issues such as insufficient coordination of care, access barriers, poor professional communication, and the lack of involvement in decisions [79]. Our results suggest that in addition to being a resource for positive experiences, high eHealth literacy may develop as a response to mitigate negative experiences with care or unfavorable health outcomes. However, these links are yet to be elucidated.

Our study was conducted in the general population without focusing on any particular disease area. Most of the respondents reported on the last ambulatory visit at their usual HCP, therefore our results reflect the general experiences of individuals with outpatient care, regardless of the nature, number, or severity of their health conditions. We applied Hungarian versions of validated instruments that have been used widely in multiple countries, such as the eHEALS or the OECD’s PREM questionnaire. We demonstrated that the composite PREM scores used in our analyses had adequate psychometric properties. However, caution is needed when generalizing our findings beyond the Hungarian setting, due to the differences of health systems, communication culture, or economic status of countries.

Furthermore, a number of limitations of our study have to be highlighted. First, only a small part of the variance of PREM items was explained by our OLS models, suggesting that potentially important determinants of patient experiences remained unexplored in our study. Moreover, eHEALS quartiles were jointly significant predictors only in case of a single PREM item, whereas—despite significant differences between the first and third quartiles—the joint test of eHEALS was not significant in 2 items. Applying refined analytical methods on a larger sample may explain patient-, physician-, and system-related factors that shape patients’ experiences of care and also clarify the relationship between unmet medical needs and eHealth literacy, which yielded mixed results in our study. A further limitation of our study is the wide recall period spanning up to 1 year between the survey and the last patient visit. Recall bias has been reported in connection with patient-experience surveys, raising concerns about the comparability between data collected with different recall periods [80]. Although recall bias of responses cannot be excluded in our study, the correlation between the time of last visit and PREM responses was minimal, suggesting negligible influence of recall bias on our results.

The potential of eHealth to improve the efficiency of health systems has been recognized by policymakers. Low health literacy is a barrier to efficient implementation of eHealth interventions [81]. eHealth literacy is viewed as resource for patients to achieve better health outcomes and participate efficiently in health production [15-21], and it can be modified with appropriate interventions [19,23]. However, in accordance with recent systematic reviews, we emphasize that the causal link between eHealth literacy and favorable patient outcomes related to health status, risk behaviors, or experiences with the health care system has not been established yet [31]. Until robust methods and clear causal links are in place, we suggest caution when implementing large-scale public health interventions based on overoptimistic expectations. Although low eHealth literacy was associated with the presence of chronic conditions, its association with a number of health-related outcomes and health behaviors has been mixed [22,31]. Our study draws the attention on another potential risk group: individuals who rate their eHealth literacy in the highest range. We found that high eHealth literacy levels were associated with both positive and negative patient experiences, a relationship which requires further exploration. Understanding the drivers of inferior experiences may help to design efficient eHealth literacy interventions, which provide individuals with resilience to navigate the health system efficiently, enable them to engage in productive partnership with HCPs, and ultimately turn online information into better health outcomes and satisfactory patient experience.

As a conclusion, our results suggest that eHealth literacy, a modifiable patient-related factor [19,23], is associated with PREMs. It is tempting to develop interventions that develop eHealth literacy along with the eHealth infrastructure and eHealth interventions aiming for better patient experiences. However, further studies are needed to establish the causal relationship between eHealth literacy and patient-reported experiences, with special focus on vulnerable individuals with low eHealth literacy levels.

## Appendix

Multimedia Appendix 1 eHealth Literacy Scale (eHEALS).

Multimedia Appendix 2 OECD-proposed Set of Questions on Patient Experiences with Ambulatory Care (PREM).

Multimedia Appendix 3 Logistic regression analysis of unmet medical needs.

Multimedia Appendix 4 Regression analysis of waiting times.

Multimedia Appendix 5 Regression analyses of unmet medical needs and waiting times PREM scores.

Multimedia Appendix 6 Sensitivity analysis.

Multimedia Appendix 7 Analysis of unmet medical needs on extended sample.
