# Supplementary material for: Exploring eHealth Literacy and Patient-Reported Experiences With Outpatient Care in the Hungarian General Adult Population: Cross-Sectional Study
Source: J Med Internet Res. 2020 Aug 11;22(8):e19013. doi: 10.2196/19013 (PMC7448194; doi:10.2196/19013)
Supplement: Multimedia Appendix 1 [file jmir_v22i8e19013_app1.pdf]

Zrubka Z, Brito Fernandes O, Baji P, Hajdu O, Kovács L, Kringos D; Klazinga N, Gulácsi L, Brodszky V, Rencz F, Péntek M. eHealth Literacy and Patient-Reported Experiences with Outpatient Care in the Hungarian General Adult Population: A Cross-Sectional Survey Study. *J Med Internet Res*. 2020

Multimedia Appendix 1

© Cameron D. Norman, Harvey A. Skinner
Used with permission under the Creative Commons 2.0 license (http://www.creativecommons.org/licenses/by/2.0/)
Norman CD, Skinner HA. eHEALS: The eHealth Literacy Scale. *J Med Internet Res*. 2006;8(4):e27. Published 2006 Nov 14. doi:10.2196/jmir.8.4.e27

I would like to ask you for your opinion and about your experience using the Internet for health information. For each statement, tell me which response best reflects your opinion and experience *right now*.

1. How **useful** do you feel the Internet is in helping you in making decisions about your health?

❑1	❑2	❑3	❑4	❑5
Not useful at all	Not useful	Unsure	Useful	Very Useful

2. How **important** is it for you to be able to access health resources on the Internet?

❑1	❑2	❑3	❑4	❑5
Not important at all	Not important	Unsure	Important	Very important

3. I know **what** health resources are available on the Internet

1) ❑ Strongly Disagree
2) ❑ Disagree
3) ❑ Undecided
4) ❑ Agree
5) ❑ Strongly Agree

4. I know **where** to find helpful health resources on the Internet

1) ❑ Strongly Disagree
2) ❑ Disagree
3) ❑ Undecided
4) ❑ Agree
5) ❑ Strongly Agree

5. I know **how** to find helpful health resources on the Internet

1) ❑ Strongly Disagree
2) ❑ Disagree
3) ❑ Undecided
4) ❑ Agree
5) ❑ Strongly Agree

6. I know **how to use** the Internet to answer my questions about health

1) ❑ Strongly Disagree
2) ❑ Disagree
3) ❑ Undecided
4) ❑ Agree
5) ❑ Strongly Agree

7. I know how to use **the health information** I find on the Internet to help me

1) ❑ Strongly Disagree
2) ❑ Disagree
3) ❑ Undecided
4) ❑ Agree
5) ❑ Strongly Agree

8. I have the skills I need to **evaluate** the health resources I find on the Internet

1) ❑ Strongly Disagree
2) ❑ Disagree
3) ❑ Undecided
4) ❑ Agree
5) ❑ Strongly Agree

9. I can tell **high quality** health resources from **low quality** health resources on the Internet

1) ❑ Strongly Disagree
2) ❑ Disagree
3) ❑ Undecided
4) ❑ Agree
5) ❑ Strongly Agree

10. I feel **confident** in using information from the Internet to make health decisions

1) ❑ Strongly Disagree
2) ❑ Disagree
3) ❑ Undecided
4) ❑ Agree
5) ❑ Strongly Agree

Thank you!

** Note: Questions #1 and #2 are recommended as supplementary items for use with the eHEALS to understand consumer's interest in using eHealth in general. These items are not a formal part of the eHealth Literacy scale, which comprises questions #3-10.*

Magyar nyelvű Elektronikus Egészségműveltség Skála (Hungarian version of the eHealth Literacy Scale, eHEALS)

© Zrubka Zsombor, Hajdu Ottó, Rencz Fanni, Baji Petra, Gulácsi László, Péntek Márta
Szabadon felhasználható a Creative Commons Attribution 4.0 International License (https://creativecommons.org/) értelmében a doi:10.1007/s10198-019-01062-1 azonosítóval ellátott közlemény referenciájának pontos megjelölésével.

A következő kérdésekkel az egészséggel kapcsolatos internethasználatról szeretnénk a véleményét és a tapasztalatait megismerni. Kérjük, mindegyik kérdésnél azt a választ jelölje, amelyik a *jelen pillanatban* a leginkább jellemző Önre.

1. Mennyire jelent **hasznos** segítséget Önnek az internet az egészségét érintő döntések során?

☐ 1	☐ 2	☐ 3	☐ 4	☐ 5
Egyáltalán nem hasznos	Nem hasznos	Nem tudom	Hasznos	Nagyon hasznos

2. Mennyire **fontos** Önnek, hogy hozzáférjen egészséggel kapcsolatos információforrásokhoz az interneten?

☐ 1	☐ 2	☐ 3	☐ 4	☐ 5
Egyáltalán nem fontos	Nem fontos	Nem tudom	Fontos	Nagyon fontos

3. Tudom, hogy **milyen** egészséggel kapcsolatos információforrások érhetők el az interneten

1) ☐ Egyáltalán nem értek egyet
2) ☐ Nem értek egyet
3) ☐ Egyet is értek meg nem is
4) ☐ Egyetértek
5) ☐ Teljesen egyetértek

4. Tudom, hogy **hol** találhatók az interneten az egészséggel kapcsolatos hasznos információforrások.

1) ☐ Egyáltalán nem értek egyet
2) ☐ Nem értek egyet
3) ☐ Egyet is értek meg nem is
4) ☐ Egyetértek
5) ☐ Teljesen egyetértek

5. Tudom, **hogyan** kell az interneten keresni az egészséggel kapcsolatos hasznos információforrásokat.

1) ☐ Egyáltalán nem értek egyet
2) ☐ Nem értek egyet
3) ☐ Egyet is értek meg nem is
4) ☐ Egyetértek
5) ☐ Teljesen egyetértek

6. Tudom, **hogyan használjam** az internetet, ha az egészséggel kapcsolatos kérdéseimet akarom megválaszolni

1) ☐ Egyáltalán nem értek egyet
2) ☐ Nem értek egyet
3) ☐ Egyet is értek meg nem is
4) ☐ Egyetértek
5) ☐ Teljesen egyetértek

7. Tudom, **hogyan hasznosítsam** az interneten talált egészséggel kapcsolatos információkat

1) ☐ Egyáltalán nem értek egyet
2) ☐ Nem értek egyet
3) ☐ Egyet is értek meg nem is
4) ☐ Egyetértek
5) ☐ Teljesen egyetértek

8. Megvan a szükséges tudásom, hogy **minősítsem** az interneten talált egészséggel kapcsolatos információforrásokat.

1) ☐ Egyáltalán nem értek egyet
2) ☐ Nem értek egyet
3) ☐ Egyet is értek meg nem is
4) ☐ Egyetértek
5) ☐ Teljesen egyetértek

9. Meg tudom egymástól különböztetni az interneten található **jó és rossz minőségű** egészséggel kapcsolatos információforrásokat

1) ☐ Egyáltalán nem értek egyet
2) ☐ Nem értek egyet
3) ☐ Egyet is értek meg nem is
4) ☐ Egyetértek
5) ☐ Teljesen egyetértek

10. Úgy érzem, **magabiztosan** használom az internetről származó információkat az egészséggel kapcsolatos döntéseim során

1) ☐ Egyáltalán nem értek egyet
2) ☐ Nem értek egyet
3) ☐ Egyet is értek meg nem is
4) ☐ Egyetértek
5) ☐ Teljesen egyetértek

Köszönjük!

** Megjegyzés: az 1. és 2. kérdések az eHEALS kiegészítői annak érdekében, hogy megértsük a válaszadó e-egészségüggyel kapcsolatos általános érdeklődését. Ezek a kérdések nem képezik az eHEALS részét, amely a 3-10. kérdésekből áll.*
