# Supplementary material for: Exploring eHealth Literacy and Patient-Reported Experiences With Outpatient Care in the Hungarian General Adult Population: Cross-Sectional Study
Source: J Med Internet Res. 2020 Aug 11;22(8):e19013. doi: 10.2196/19013 (PMC7448194; doi:10.2196/19013)
Supplement: Multimedia Appendix 2 [file jmir_v22i8e19013_app2.pdf]

Zrubka Z, Brito Fernandes O, Baji P, Hajdu O, Kovács L, Kringos D; Klazinga N, Gulácsi L, Brodszky V, Rencz F, Péntek M. eHealth Literacy and Patient-Reported Experiences with Outpatient Care in the Hungarian General Adult Population: A Cross-Sectional Survey Study. *J Med Internet Res*. 2020

Multimedia Appendix 2

OECD-proposed Set of Questions on Patient Experiences with Ambulatory Care
Adapted with permission for online research. Source: Fujisawa, R. and S.N. Klazinga, *Measuring patient experiences (PREMS): Progress made by the OECD and its member countries between 2006 and 2016*. 2017: Paris.

We would like to ask you a few questions about your experiences with access to and use of outpatient health care over the past 12 months.

Access to care

Q4 When was the last time that you had a consultation with a doctor, nurse or other health professional to get care for yourself? Include both consultations over the phone and consultations in a doctor's office, a clinic, or the outpatient department of a hospital
Do not include:

- care you got when you stayed overnight in a hospital.
- times you went for dental care visits.
- accident and emergency care or
- care received in your home.

1 In the last 30 days
2 Between 1 and 3 months ago
3 More than 3 but less than 6 months ago
4 Between 6 and 12 months ago
5 Between one and two years ago → *Ask Q5-Q9, then go to Q21*
6 More than two years ago → *Ask Q5-Q9, then go to Q21*
7 I'm not sure → *Ask Q6-Q9, then go to Q21*
8 I do not want to answer → *Ask Q6-Q9, then go to Q21*

Q5 Was it at a GP/family practice, health centre, or clinic that you usually go to for most of your medical care?
1 Yes
2 No, not at my usual place for medical care
3 No, do not have a usual place for medical care
4 I'm not sure
5 I do not want to answer

Q6 During the last 12 months, was there a time when you had a medical problem but did not visit a doctor, nurse or other health professional because of difficulties in travelling to (getting to) a doctor's office, clinic or the outpatient department of a hospital?
1 Yes
2 No
3 Does not apply to me, I did not have health problems over the past 12 months → *if Q4=1-4, go to Q9, if Q4=5-8, then go to END*
[Depending on the answer to Q4]
4 I'm not sure
5 I do not want to answer

Q7 During the last 12 months, was there a time when you had a medical problem but did not visit a doctor, nurse or other health professional because of cost? (Think about expenses that you pay for your care.)

1 Yes
2 No
3 Not applicable
4 I'm not sure
5 I do not want to answer

Q8 During the last 12 months, was there a time when you skipped a medical test, treatment (excluding medicines), or other follow-up that was recommended by a doctor, nurse or other health professional because of the cost? (Think about expenses that you pay for your care.)

1 Yes
2 No
3 Not applicable
4 I'm not sure
5 I do not want to answer

Q9 In the last 12 months, was there a time when you did not fill a prescription for medicine/collect a prescription for medicine, or you skipped doses of your medicine because of the cost? (Think about expenses that you pay for your medicines.)

1 Yes
2 No
3 Not applicable
4 I'm not sure
5 I do not want to answer

I now want to ask some questions about the last time that you had a consultation with a doctor, nurse or other health professional to get care for yourself. This can be a consultation over the phone or a consultation in a doctor's office, a clinic, or the outpatient department of a hospital.
Do not include:

- care you got when you stayed overnight in a hospital.
- times you went for dental care visits.
- accident and emergency care or
- care received in your home.

Q10 Thinking about this last consultation, which of the following best describes the type of care you principally received? If more than one applies, please choose the one that was the main type of care. Please consider heatlhcare professionals working in mental health care as well.

1 General practitioner / family physician at a doctor's office
2 Specialist at an outpatient department of a state hospital
3 Specialist at a state outpatient care office
4 Specialist at a private doctor's office
3 Nurse at outpatient department of a state hospital
3 Nurse at a state outpatient care office
4 Nurse at a private doctor's office
5 Nurse at a community based clinic
6 Other health professional at outpatient department of a hospital
7 Other health professional at a doctor's office
8 Other health professional at a community based clinic
9 Telephone consultation either by general practitioner/family physician, specialist, nurse, or other health professional → *skip to Q15*

Q11 How quickly did you get an appointment to this consultation (visit, checkup)?

1 Same day → *skip to Q13*
2 Next day
3 2 to 5 days (≈ couple of days)
4 6 to 7 days (≈just less than a week)
5 8 to 14 days (≈ more than 1 week)
6 15 to 30 days (≈ more than 2 weeks)
7 31 to 60 days (≈ more than 1 month)
8 61 to 90 days (≈ more than 2 months)
9 91 days or longer (≈ more than 3 months)
10 I did not make an appointment, I went directly to the doctor/nurse → *skip to Q13*
11 I'm not sure
12 I do not want to answer

Q12 Was the time you waited for the appointment a problem for you?

1 Yes
2 No

Q13 On the actual day of the consultation, how long did you wait (for example in the doctor's waiting room) before you were actually seen?

1 Up to 15 minutes → *skip to Q15*
2 More than 15 and up to 30 minutes (≈up to half an hour)
3 More than 30 and up to 60 minutes (≈up to an hour)
4 More than 1 and up to 2 hours
5 More than 2 and up to 4 hours
6 More than 4 and up to 8 hours
7 More than 8 hours
8 I left before seeing the healthcare provider that day → *skip to Q15*
9 I'm not sure
10 I do not want to answer

Q14 Was the time you waited to be seen a problem for you?

1 Yes
2 No

Patient experiences

Now, the following questions still refer to the last time you had a consultation with a doctor, nurse or other health professional. In the questions we will refer as *health professional* to the doctor, nurse or other health professional whom you visited last time.

Q15 Did this health professional spend enough time with you?

1 Yes, definitely
2 Yes, to some extent
3 No, not really
4 No, definitely not
5 I'm not sure
6 I do not want to answer

Q16 Did this health professional explain things in a way that was easy to understand?

1 Yes, definitely
2 Yes, to some extent
3 No, not really
4 No, definitely not
5 I'm not sure
6 I do not want to answer

Q17 Did this health professional give you an opportunity to ask questions or raise concerns about recommended treatment?

1 Yes, definitely
2 Yes, to some extent
3 No, not really
4 No, definitely not
5 I'm not sure
6 I do not want to answer

Q18 Did this health professional involve you as much as you wanted to be in decisions about your care and treatment?

1 Yes, definitely
2 Yes, to some extent
3 No, not really
4 No, definitely not
5 No, did not want to be involved
6 Not applicable: no decisions about treatment were made
7 I'm not sure
8 I do not want to answer

Q19 Overall, how would you rate the quality of this consultation?

1 Excellent
2 Very good
3 Good
4 Fair
5 Poor
6 I'm not sure
7 I do not want to answer
