# Supplementary material for: Exploring eHealth Literacy and Patient-Reported Experiences With Outpatient Care in the Hungarian General Adult Population: Cross-Sectional Study
Source: J Med Internet Res. 2020 Aug 11;22(8):e19013. doi: 10.2196/19013 (PMC7448194; doi:10.2196/19013)
Supplement: Multimedia Appendix 3 [file jmir_v22i8e19013_app3.pdf]

Zrubka Z, Brito Fernandes O, Baji P, Hajdu O, Kovács L, Kringos D; Klazinga N, Gulácsi L, Brodszky V, Rencz F, Péntek M. eHealth Literacy and Patient-Reported Experiences with Outpatient Care in the Hungarian General Adult Population: A Cross-Sectional Survey Study. *J Med Internet Res*. 2020

Multimedia Appendix 3

Logistic regression analysis of unmet medical needs

	**Travel**[a]		**Visit**[b]		**Intervention**[c]		**Medication**[d]	
	Beta	*P* value	Beta	*P* value	Beta	*P* value	Beta	*P* value
**eHEALS**[e]								
2nd quartile	-0.19	.57	-0.33	.37	-0.42	.27	0.11	.74
3rd quartile	-0.62	.05	-0.15	.66	-0.75	.05	-0.19	.56
4th quartile	-0.24	.46	-0.17	.66	-0.07	.85	-0.31	.39
**Age group**[f]								
25-44 years old	0.25	.56	0.37	.45	0.89	.14	-0.32	.46
45-64 years old	-0.05	.91	-0.41	.46	-0.13	.85	-0.81	.1
65+ years old	-0.30	.57	-0.36	.54	0.35	.61	-0.98	.06
**Education**[g]								
Secondary	0.22	.45	0.24	.46	-0.16	.64	0.37	.21
Tertiary	-0.84	.02	-0.67	.09	-0.55	.16	-1.00	.009
**Gender**								
Male	-0.36	.16	-0.32	.26	-0.02	.94	-0.39	.14
**Income**[h]								
2nd quintile	-0.47	.18	-0.22	.57	0.31	.44	0.16	.65
3rd quintile	-1.14	.01	-1.02	.06	-0.46	.38	0.07	.87
4th quintile	-0.43	.26	-0.17	.69	-0.30	.52	-0.03	.95
5th quintile	-0.61	.08	-0.59	.14	-0.50	.23	-0.65	.09
**Paid employment**								
Yes	-0.21	.46	0.03	.93	-0.05	.88	0.05	.86
**Family status**								
Married / domestic partnership	0.43	.08	0.04	.89	0.35	.22	0.10	.69
**Residence**[i]								
City	-0.17	.58	-0.57	.08	0.51	.18	-0.20	.54
Village	-0.02	.97	-0.43	.27	0.06	.9	-0.36	.35
**Self-perceived health**[j]								
Very bad	1.50	.29	-0.37	.8	1.41	.35	0.82	.56
Bad	1.55	.03	0.38	.6	1.65	.046	0.22	.75
Fair	0.68	.25	-0.32	.61	1.04	.15	0.22	.7
Good	0.36	.51	-0.08	.89	0.46	.5	-0.55	.31
**GALI**[k]								
Limited but not severely	0.40	.15	0.66	.04	0.10	.75	1.03	<.001
Severely limited	0.78	.09	1.05	.04	0.43	.4	1.60	<.001
**Chronic morbidity**								
Yes	0.15	.63	0.75	.04	0.22	.57	0.16	.63
**Setting**[l]								
Public specialist	0.03	.95	0.99	.08	0.29	.63	0.17	.77
Private specialist	0.82	.19	1.98	.002	0.91	.19	-0.05	.94
**HCP type**[m]								
Specialist	-0.36	.49	-1.31	.02	-0.12	.83	-0.16	.77
Other	-	-	-	-	-	-	-	-
**Usual HCP**								
Yes	0.26	.45	0.06	.87	0.04	.93	0.02	.95
**Constant**	-1.23	.14	-1.29	.16	-3.20	.002	-0.71	.39
n	497		498		501		499	
LR[n] test Chi-square (28)	77.8	<0.001	63.6	<0.001	46.5	.02	93.5	<0.001
GOF[o] test Chi-square (462)	492.5	.16						
GOF test Chi-square (457)			518.2	.04				
GOF test Chi-square (458)					505.3	.10		
GOF test Chi-square (456)							488.1	.21

[a]Missed visit due to travel burden
[b]Missed visit due to cost burden
[c]Missed intervention due to cost burden
[d]Missed medication due to cost burden
[e]Base: 1st quartile
[f]Base: 18-24 years old
[g]Base: Primary

[h]Base: 1st quintile
[i]Base: Capital
[j]Base: Very good
[k]Base: Not limited
[l]Base: General practitioner
[m]Base: General practitioner
[n]Likelihood ratio; omnibus test for independence, current model versus null model
[o]Goodness of fit; Hosmer-Lemeshow test
