# Supplementary material for: Exploring eHealth Literacy and Patient-Reported Experiences With Outpatient Care in the Hungarian General Adult Population: Cross-Sectional Study
Source: J Med Internet Res. 2020 Aug 11;22(8):e19013. doi: 10.2196/19013 (PMC7448194; doi:10.2196/19013)
Supplement: Multimedia Appendix 4 [file jmir_v22i8e19013_app4.pdf]

Zrubka Z, Brito Fernandes O, Baji P, Hajdu O, Kovács L, Kringos D; Klazinga N, Gulácsi L, Brodszky V, Rencz F, Péntek M. eHealth Literacy and Patient-Reported Experiences with Outpatient Care in the Hungarian General Adult Population: A Cross-Sectional Survey Study. *J Med Internet Res*. 2020

Multimedia Appendix 4

Regression analysis of waiting times

	**oWP[a]**		**aWP[b]**		**Log-oWT[c]**		**Log-aWT[d]**	
Model	Logistic		Logistic		OLS[n]		OLS[n]	
	Beta	*P* value	Beta	*P* value	Beta	*P* value	Beta	*P* value
**eHEALS[e]**								
2nd quartile	0.24	.45	-0.48	.22	0.09	.54	0.03	.89
3rd quartile	0.34	.27	-0.34	.35	0.04	.78	-0.03	.87
4th quartile	0.46	.15	-0.32	.41	0.13	.38	0.02	.91
**Age group[f]**								
25-34 years old	0.03	.95	-0.35	.52	0.14	.49	-0.09	.76
45-64 years old	-0.78	.11	-0.46	.45	-0.06	.78	0.12	.70
65+ years old	-0.72	.17	-0.77	.24	0.08	.74	0.32	.34
**Education[g]**								
Secondary	-0.17	.55	-0.33	.36	-0.07	.58	-0.22	.23
Tertiary	-0.17	.60	-0.20	.61	-0.18	.19	-0.10	.63
**Gender**								
Male	-0.07	.78	-0.89	.004	0.05	.64	-0.07	.63
**Income[h]**								
2nd quintile	-0.36	.30	0.46	.29	0.04	.78	-0.18	.43
3rd quintile	-0.95	.03	0.55	.26	-0.30	.11	0.12	.68
4th quintile	-0.43	.27	-0.25	.65	0.06	.71	-0.15	.54
5th quintile	-0.26	.45	0.30	.49	-0.16	.30	-0.32	.16
**Paid employment**								
Yes	-0.18	.54	-0.18	.62	-0.09	.46	0.10	.58
**Family status**								
Married / domestic partnership	-0.47	.06	-0.12	.68	-0.03	.76	-0.19	.22
**Residence[i]**								
City	0.34	.26	0.43	.27	0.20	.12	-0.15	.43
Village	0.03	.94	0.19	.68	0.03	.85	-0.41	.07
**Self-perceived health[j]**								
Very bad	2.55	.08	-	-	-0.01	.99	-1.18	.24
Bad	1.34	.04	0.21	.78	0.45	.11	0.35	.39
Fair	0.71	.18	0.34	.61	0.23	.29	0.31	.33
Good	0.61	.22	-0.18	.77	0.27	.18	0.13	.65
**GALI[k]**								
Limited but not severely	0.66	.01	0.64	.05	0.33	.005	-0.06	.74
Severely limited	0.49	.30	1.19	.02	0.33	.13	0.08	.82
**Chronic morbidity**								
Yes	-	.99	0.15	.70	0.03	.80	0.11	.58
**Setting[l]**								
Public specialist	-0.37	.55	0.92	.18	0.52	.04	1.38	<.001
Private specialist	-0.70	.32	1.15	.13	-0.30	.31	1.02	.02
**HCP type[m]**								
Specialist	0.13	.83	0.37	.57	-0.37	.14	0.71	.05
Other	-	-	-	-	-	-	-	-
**Usual HCP**								
Yes	0.56	.11	0.80	.06	-	.97	-0.21	.31
**Constant**	-1.81	.03	-3.04	.003	3.07	<.001	0.45	.38
n	505		502		502		492	
LR[o] test Chi-square (28)	52.2	.004	63.8	<.001				
LR test F(28;473)					2.49	<.001		
LR test F(28;463)							8.92	<.001
R^2					0.13		0.31	
GOF[p] test Chi-square (470)	488.1	.27						
GOF[p] test Chi-square (468)			515.0	.07				
Breusch-Pagan test Chi-square (1)					0.9	.33	1.9	.16
Ramsey RESET test F(3;470)					0.8	.49		
Ramsey RESET test F(3;460)							4.3	.005

[a]Office waiting time was a problem

[b]Appointment waiting time was a problem
[c]Log-office waiting time
[d]Log- appointment waiting time
[e]Base: 1st quartile
[f]Base: 18-24 years old
[g]Base: Primary
[h]Base: 1st quintile
[i]Base: Capital
[j]Base: Very good
[k]Base: Not limited
[l]Base: General practitioner
[m]Base: General practitioner
[n]OLS: Ordinary least squares regression
[o]Likelihood ratio; omnibus test for independence, current model versus null model
[p]Goodness of fit; Hosmer-Lemeshow test
