# Supplementary material for: Exploring eHealth Literacy and Patient-Reported Experiences With Outpatient Care in the Hungarian General Adult Population: Cross-Sectional Study
Source: J Med Internet Res. 2020 Aug 11;22(8):e19013. doi: 10.2196/19013 (PMC7448194; doi:10.2196/19013)
Supplement: Multimedia Appendix 5 [file jmir_v22i8e19013_app5.pdf]

Zrubka Z, Brito Fernandes O, Baji P, Hajdu O, Kovács L, Kringos D; Klazinga N, Gulácsi L, Brodszky V, Rencz F, Péntek M. eHealth Literacy and Patient-Reported Experiences with Outpatient Care in the Hungarian General Adult Population: A Cross-Sectional Survey Study. *J Med Internet Res*. 2020

Multimedia Appendix 5

Regression analyses of unmet medical needs and waiting times PREM scores

	**Square root Unmet Medical Needs Score**		**Any Unmet Medical Needs**		**Any Waiting Problem**	
Model	Robust[p]		Logistic		Logistic	
	Beta	*P* value	Beta	*P* value	Beta	*P* value
**eHEALS[e]**						
2nd quartile	-0.05	.58	-0.08	.79	-0.02	.94
3rd quartile	-0.15	.06	-0.57	.05	0.23	.42
4th quartile	-0.08	.38	-0.41	.19	0.07	.81
**Age group[f]**						
25-44 years old	-0.09	.41	-0.84	.05	-0.31	.45
45-64 years old	-0.29	.02	-1.45	.002	-0.96	.03
65+ years old	-0.26	.06	-1.27	.01	-1.34	.007
**Education[g]**						
Secondary	0.05	.52	0.12	.66	-0.31	.25
Tertiary	-0.24	.006	-0.87	.005	-0.22	.46
**Gender**						
Male	-0.11	.1	-0.31	.19	-0.52	.02
**Income[h]**						
2nd quintile	-0.03	.78	-0.16	.64	-0.08	.82
3rd quintile	-0.23	.04	-0.72	.08	-0.45	.26
4th quintile	-0.10	.39	-0.29	.42	-0.24	.52
5th quintile	-0.24	.02	-0.83	.01	-0.05	.89
**Paid employment**						
Yes	-0.05	.51	-0.34	.23	-0.21	.44
**Family status**						
Married / domestic partnership	0.07	.28	0.26	.26	-0.45	.05
**Residence[i]**						
City	-0.08	.27	-0.37	.19	0.49	.09
Village	-0.03	.78	0.05	.89	0.31	.37
**Self-perceived health[j]**						
Very bad	0.64	.009	-	-	1.77	.21
Bad	0.45	.01	1.82	.006	1.15	.06
Fair	0.21	.13	1.15	.04	0.76	.13
Good	0.11	.39	0.81	.11	0.40	.39
**GALI[k]**						
Limited but not severely	0.17	.02	0.50	.04	0.72	.003
Severely limited	0.33	.04	0.91	.049	0.58	.19
**Chronic morbidity**						
Yes	0.16	.04	0.45	.12	0.33	.25
**Setting[l]**						
Public specialist	0.22	.22	0.74	.21	-0.07	.9
Private specialist	0.43	.03	1.21	.08	-0.34	.59
**HCP type[m]**						
Specialist	-0.27	.12	-0.74	.21	0.29	.6
Other	-	-	-	-	-	-
**Usual HCP**						
Yes	0.05	.54	0.37	.23	0.72	.02
**Constant**	0.66	.002	-0.03	.97	-1.56	.04
n	484		481		505	
LR[n] test F(28;455)	7.15	<.001				
LR test Chi-square (27)			114.1	<.001		
LR test Chi-square (28)					74.8	<.001
R^2	0.23					
Ramsey RESET test F(3;452)	1.77	.15				
GOF[o] test Chi-square (447)			488.9	.08		
GOF test Chi-square (462)					482.8	.33

[a]Office waiting time was a problem

[b]Appointment waiting time was a problem
[c]Log-office waiting time
[d]Log- appointment waiting time
[e]Base: 1st quartile
[f]Base: 18-24 years old
[g]Base: Primary
[h]Base: 1st quintile
[i]Base: Capital
[j]Base: Very goodr
[k]Base: Not limited
[l]Base: General practitioner
[m]Base: General practitioner
[n]Likelihood ratio; omnibus test for independence, current model versus null model
[o]Goodness of fit, Hosmer-Lemeshow test
[p]Ordinary least squares regression with robust standard errors
