# Supplementary material for: Exploring eHealth Literacy and Patient-Reported Experiences With Outpatient Care in the Hungarian General Adult Population: Cross-Sectional Study
Source: J Med Internet Res. 2020 Aug 11;22(8):e19013. doi: 10.2196/19013 (PMC7448194; doi:10.2196/19013)
Supplement: Multimedia Appendix 6 [file jmir_v22i8e19013_app6.pdf]

Zrubka Z, Brito Fernandes O, Baji P, Hajdu O, Kovács L, Kringos D; Klazinga N, Gulácsi L, Brodszky V, Rencz F, Péntek M. eHealth Literacy and Patient-Reported Experiences with Outpatient Care in the Hungarian General Adult Population: A Cross-Sectional Survey Study. J Med Internet Res. 2020

Multimedia Appendix 6. Sensitivity analysis of PREM[a] regression models by varying eHEALS[b] group category boundaries

Scenario		eHEALS Group 1	eHEALS Group 2	eHEALS Group 3	eHEALS Group 4
#1	eHEALS score range	0-25	26-28	29-32	33-40
	N (%)	148 (22.2)	126 (18.9)	250 (37.5)	142 (21.3)
#2	eHEALS score range	0-25	26-28	29-34	35-40
	N (%)	148 (22.2)	126 (18.9)	299 (44.9)	93 (14.0)
#3	eHEALS score range	0-25	26-29	30-32	33-40
	N (%)	148 (22.2)	194 (29.1)	182 (27.3)	142 (21.3)
#4	eHEALS score range	0-25	26-29	30-34	35-40
	N (%)	148 (22.2)	194 (29.1)	231 (34.7)	93 (14.0)
#5	eHEALS score range	0-25	26-30	31-32	33-40
	N (%)	148 (22.2)	235 (35.3)	141 (21.2)	142 (21.3)
#6	eHEALS score range	0-25	26-30	31-34	35-40
	N (%)	148 (22.2)	235 (35.3)	190 (28.5)	93 (14.0)
#7	eHEALS score range	0-26	27-28	29-32	33-40
	N (%)	191 (28.7)	83 (12.5)	250 (37.5)	142 (21.3)
#8	eHEALS score range	0-26	27-28	29-34	35-40
	N (%)	191 (28.7)	83 (12.5)	299 (44.9)	93 (14.0)
#9	eHEALS score range	0-26	27-29	30-32	33-40
	N (%)	191 (28.7)	151 (22.7)	182 (27.3)	142 (21.3)
#10	eHEALS score range	0-26	27-29	30-34	35-40
	N (%)	191 (28.7)	151 (22.7)	231 (34.7)	93 (14.0)
#11	eHEALS score range	0-26	27-30	31-32	33-40
	N (%)	191 (28.7)	192(28.8)	141 (21.2)	142 (21.3)
#12	eHEALS score range	0-26	27-30	31-34	35-40
	N (%)	191 (28.7)	192(28.8)	190 (28.5)	93 (14.0)

[a]PREM: OECD-proposed Set of Questions on Patients' Experiences with Ambulatory Care; [b]eHealth Literacy Scale

Time[a]		Scenario	#1	#2	#3	#4	#5	#6	#7	#8	#9	#10	#11	#12
	Sample	N	502	502	502	502	502	502	502	502	502	502	502	502
	LR test[b]	$Chi^2_{(28)}$	51.2	52.4	51.6	52.9	51.0	52.2	53.3	54.4	52.7	54.1	53.0	54.1
		P	.005	.003	.004	.003	.005	.004	.003	.002	.003	.002	.003	.002
	eHEALS Group 2 vs base	beta	0.01	0.02	0.03	0.03	-0.06	-0.05	-0.51	-0.5	-0.3	-0.3	-0.37	-0.37
		P	.97	.95	.92	.91	.82	.84	.16	.16	.28	.28	.16	.16
	eHEALS Group 3 vs base	beta	-0.1	-0.14	-0.16	-0.2	-0.08	-0.15	-0.25	-0.29	-0.31	-0.35	-0.22	-0.29
		P	.70	.58	.57	.46	.80	.60	.30	.21	.24	.16	.44	.26
	eHEALS Group 4 vs base	beta	0.00	0.2	0.00	0.2	0.00	0.2	-0.14	0.05	-0.14	0.05	-0.14	0.05
		P	.99	.55	.99	.56	.99	.56	.60	.88	.61	.88	.61	.88

Ordered logit model, the constant and coefficients of the following variables were not displayed: Age group, Education, Gender, Income, Paid employment, Family status, Residence, Self-perceived health, Activity limitations, Chronic morbidity, Setting, HCP type, Regular HCP

[a]Doctor spending enough time with patient in consultation (4-point Likert scale)

[b] Likelihood ratio; omnibus test for independence, current model versus null model

[c] base: eHEALS Group 1

**Understand**[a]		**Scenario**	**#1**	**#2**	**#3**	**#4**	**#5**	**#6**	**#7**	**#8**	**#9**	**#10**	**#11**	**#12**
	Sample	N	504	504	504	504	504	504	504	504	504	504	504	504
	LR test[b]	$Chi^2_{(28)}$	55.0	56.2	59.8	61.0	59.7	60.9	56.3	57.5	60.6	61.9	61.0	62.2
		P	.002	.001	<.001	<.001	<.001	<.001	.001	<.001	<.001	<.001	<.001	<.001
	eHEALS Group 2 vs base[c]	beta	-0.16	-0.15	-0.13	-0.12	-0.21	-0.2	-0.43	-0.42	-0.29	-0.28	-0.36	-0.36
		P	.62	.64	.66	.66	.44	.45	.22	.23	.31	.32	.17	.17
	eHEALS Group 3 vs base	beta	-0.66	-0.68	-0.93	-0.9	-1.06	-0.99	-0.72	-0.74	-0.98	-0.95	-1.11	-1.04
		P	.02	.01	.003	.002	.002	.001	.005	.003	<.001	<.001	<.001	<.001
	eHEALS Group 4 vs base	beta	-0.44	-0.25	-0.45	-0.27	-0.46	-0.26	-0.5	-0.31	-0.51	-0.32	-0.51	-0.32
		P	.16	.48	.15	.45	.15	.45	.09	.36	.09	.34	.09	.34

Ordered logit model, the constant and coefficients of the following variables were not displayed: Age group, Education, Gender, Income, Paid employment, Family status, Residence, Self-perceived health, Activity limitations, Chronic morbidity, Setting, HCP type, Regular HCP

[a] Doctor providing easy to understand explanations (4-point Likert scale)

[b] Likelihood ratio; omnibus test for independence, current model versus null model

[c] base: eHEALS Group 1

Questions[a]		Scenario	#1	#2	#3	#4	#5	#6	#7	#8	#9	#10	#11	#12
	Sample	N	500	500	500	500	500	500	500	500	500	500	500	500
	LR test[b]	$Chi^2_{(28)}$	42.1	42.6	41.5	42.0	41.4	41.8	43.6	44.1	43.7	44.2	43.9	44.3
		P	.04	.04	.049	.04	.049	.04	.03	.03	.03	.03	.03	.03
	eHEALS Group 2 vs base[c]	beta	0.00	0.00	-0.11	-0.1	-0.14	-0.14	-0.42	-0.41	-0.42	-0.42	-0.42	-0.42
		P	.99	.99	.79	.71	.59	.69	.21	.22	.12	.13	.19	.19
	eHEALS Group 3 vs base	beta	-0.38	-0.37	-0.41	-0.4	-0.46	-0.42	-0.51	-0.5	-0.54	-0.52	-0.58	-0.54
		P	.15	.14	.14	.14	.14	.13	.04	.03	.04	.03	.04	.04
	eHEALS Group 4 vs base	beta	-0.13	-0.03	-0.14	-0.03	-0.14	-0.04	-0.26	-0.16	-0.26	-0.16	-0.26	-0.16
		P	.65	.93	.64	.92	.64	.91	.34	.61	.34	.61	.34	.61

Ordered logit model, the constant and coefficients of the following variables were not displayed: Age group, Education, Gender, Income, Paid employment, Family status, Residence, Self-perceived health, Activity limitations, Chronic morbidity, Setting, HCP type, Regular HCP

[a] Doctor giving opportunity to ask questions or raise concerns (4-point Likert scale)

[b] Likelihood ratio; omnibus test for independence, current model versus null model

[c] base: eHEALS Group 1

Decisions[a]		Scenario	#1	#2	#3	#4	#5	#6	#7	#8	#9	#10	#11	#12
	Sample	N	477	477	477	477	477	477	477	477	477	477	477	477
	LR test[b]	$Chi^2_{(28)}$	50.9	48.9	50.8	48.9	50.6	49.1	50.8	48.8	50.7	48.8	50.6	49.0
		P	.005	.008	.005	.009	.006	.008	.005	.009	.005	.009	.006	.008
	eHEALS Group 2 vs base[c]	beta	-0.18	-0.18	-0.21	-0.21	-0.27	-0.27	-0.16	-0.16	-0.2	-0.2	-0.26	-0.26
		P	.56	.55	.43	.43	.31	.31	.63	.64	.47	.48	.31	.32
	eHEALS Group 3 vs base	beta	-0.32	-0.23	-0.34	-0.22	-0.3	-0.17	-0.29	-0.2	-0.3	-0.18	-0.26	-0.13
		P	.21	.34	.21	.39	.30	.53	.22	.39	.23	.43	.33	.60
	eHEALS Group 4 vs base	beta	0.11	0.06	0.11	0.06	0.11	0.06	0.15	0.1	0.15	0.1	0.15	0.1
		P	.69	.86	.70	.87	.70	.86	.57	.75	.57	.75	.57	.75

Ordered logit model, the constant and coefficients of the following variables were not displayed: Age group, Education, Gender, Income, Paid employment, Family status, Residence, Self-perceived health, Activity limitations, Chronic morbidity, Setting, HCP type, Regular HCP

[a]Doctor involving patient in decisions about care and treatment (4-point Likert scale)

[b] Likelihood ratio; omnibus test for independence, current model versus null model

[c] base: eHEALS Group 1

Overall quality[a]	Scenario	#1	#2	#3	#4	#5	#6	#7	#8	#9	#10	#11	#12
Sample	N	503	503	503	503	503	503	503	503	503	503	503	503
LR test[b]	$Chi^2_{(28)}$	39.9	39.9	41.2	40.9	41.6	41.1	40.8	40.7	42.1	41.9	42.9	42.4
	P	.07	.07	.05	.05	.047	.05	.06	.06	.04	.04	.04	.04
eHEALS Group 2 vs base[c]	beta	-0.11	-0.11	-0.06	-0.06	-0.03	-0.03	0.29	0.29	0.24	0.24	0.25	0.25
	P	.67	.67	.79	.80	.90	.90	.31	.31	.31	.31	.26	.26
eHEALS Group 3 vs base	beta	0.3	0.29	0.41	0.37	0.48	0.42	0.44	0.43	0.55	0.51	0.62	0.56
	P	.18	.19	.09	.11	.06	.08	.03	.03	.02	.02	.01	.01
eHEALS Group 4 vs base	beta	0.19	0.18	0.2	0.19	0.2	0.19	0.34	0.33	0.34	0.33	0.34	0.33
	P	.45	.52	.44	.51	.44	.51	.16	.23	.16	.22	.15	.22

Ordered logit model, the constant and coefficients of the following variables were not displayed: Age group, Education, Gender, Income, Paid employment, Family status, Residence, Self-perceived health, Activity limitations, Chronic morbidity, Setting, HCP type, Regular HCP

[a]Overall, how would you rate the quality of this consultation? (5-point Likert-scale)

[b] Likelihood ratio; omnibus test for independence, current model versus null model

[c] base: eHEALS Group 1

Log-problem score[a]	Scenario	#1	#2	#3	#4	#5	#6	#7	#8	#9	#10	#11	#12
Sample	N	473	473	473	473	473	473	473	473	473	473	473	473
LR test[b]	$F_{(28,444)}$	2.67	2.71	2.61	2.65	2.55	2.59	2.65	2.69	2.63	2.66	2.60	2.63
	P	<.001	<.001	<.001	<.001	<.001	<.001	<.001	<.001	<.001	<.001	<.001	<.001
eHEALS Group 2 vs base[c]	beta	0.01	0.01	-0.02	-0.01	-0.04	-0.04	-0.05	-0.05	-0.06	-0.06	-0.08	-0.08
	P	.90	.90	.76	.76	.45	.45	.36	.36	.23	.23	.09	.09
eHEALS Group 3 vs base	beta	-0.07	-0.07	-0.08	-0.07	-0.07	-0.06	-0.09	-0.08	-0.1	-0.09	-0.09	-0.08
	P	.11	.13	.08	.11	.15	.19	.03	.04	.02	.03	.05	.07
eHEALS Group 4 vs base	beta	0	0.02	0	0.02	0	0.02	-0.02	0	-0.02	0	-0.02	0
	P	.96	.71	.97	.72	.97	.72	.74	.96	.73	.96	.74	.95

Ordinary least squares regression (OLS) with robust standard errors, the constant and coefficients of the following variables were not displayed: Age group, Education, Gender, Income, Paid employment, Family status, Residence, Self-perceived health, Activity limitations, Chronic morbidity, Setting, HCP type, Regular HCP

[a] Problem Score: the sum of answers for each PREM item (1: yes, definitely – 4: definitely not), higher values represent more problems experienced during the visit.

[b] Likelihood ratio; omnibus test for independence, current model versus null model

[c] base: eHEALS Group 1

Negative experiences score[a]	Scenario	#1	#2	#3	#4	#5	#6	#7	#8	#9	#10	#11	#12
Sample	N	473	473	473	473	473	473	473	473	473	473	473	473
LR test[b]	$F_{(28,444)}$	2.80	2.85	2.75	2.79	2.71	2.76	2.81	2.86	2.81	2.85	2.80	2.84
	P	<.001	<.001	<.001	<.001	<.001	<.001	<.001	<.001	<.001	<.001	<.001	<.001
eHEALS Group 2 vs base[c]	beta	-0.05	-0.05	-0.16	-0.16	-0.21	-0.21	-0.32	-0.32	-0.37	-0.36	-0.4	-0.4
	P	.82	.82	.45	.45	.31	.31	.17	.17	.08	.08	.046	.046
eHEALS Group 3 vs base	beta	-0.37	-0.34	-0.38	-0.35	-0.37	-0.33	-0.44	-0.42	-0.46	-0.42	-0.44	-0.4
	P	.07	.08	.07	.09	.09	.11	.02	.02	.02	.02	.03	.03
eHEALS Group 4 vs base	beta	-0.1	-0.03	-0.1	-0.04	-0.1	-0.04	-0.17	-0.11	-0.17	-0.11	-0.17	-0.11
	P	.66	.89	.65	.88	.65	.88	.40	.64	.40	.64	.40	.64

Ordinary least squares regression (OLS) with robust standard errors, the constant and coefficients of the following variables were not displayed: Age group, Education, Gender, Income, Paid employment, Family status, Residence, Self-perceived health, Activity limitations, Chronic morbidity, Setting, HCP type, Regular HCP

[a] Negative Experiences Score: the count of PREM items that did not receive a "yes, definitely" (best experience) answer

[b] Likelihood ratio; omnibus test for independence, current model versus null model

[c] base: eHEALS Group 1

**Any negative experience[a]**	**Scenario**	**#1**	**#2**	**#3**	**#4**	**#5**	**#6**	**#7**	**#8**	**#9**	**#10**	**#11**	**#12**
Sample	N	505	505	505	505	505	505	505	505	505	505	505	505
LR test[b]	$Chi^2_{(28)}$	51.0	50.9	48.9	48.9	48.9	48.8	50.1	50.1	49.6	49.6	50.0	49.9
	P	.005	.005	.009	.009	.009	.009	.006	.006	.007	.007	.007	.007
eHEALS Group 2 vs base[c]	beta	0.3	0.3	0.04	0.04	-0.02	-0.01	-0.01	-0.01	-0.25	-0.25	-0.28	-0.28
	P	.36	.36	.89	.89	.96	.96	.98	.98	.38	.38	.29	.29
eHEALS Group 3 vs base	beta	-0.12	-0.1	-0.03	-0.03	0.04	0.02	-0.26	-0.24	-0.16	-0.17	-0.09	-0.11
	P	.67	.70	.93	.92	.89	.94	.30	.31	.54	.51	.75	.67
eHEALS Group 4 vs base	beta	-0.02	-0.02	-0.03	-0.03	-0.03	-0.03	-0.17	-0.17	-0.17	-0.16	-0.17	-0.16
	P	.94	.96	.92	.94	.92	.94	.56	.61	.56	.61	.56	.61

Logistic regression, the constant and coefficients of the following variables were not displayed: Age group, Education, Gender, Income, Paid employment, Family status, Residence, Self-perceived health, Activity limitations, Chronic morbidity, Setting, HCP type, Regular HCP

[a]Any negative experience: the response to a PREM-item was other than "yes, definitely" (best experience)

[b] Likelihood ratio; omnibus test for independence, current model versus null model

[c] base: eHEALS Group 1

Travel[a]		Scenario	#1	#2	#3	#4	#5	#6	#7	#8	#9	#10	#11	#12
	Sample	N	497	497	497	497	497	497	497	497	497	497	497	497
	LR test[b]	$Chi^2_{(28)}$	75.7	75.5	77.8	77.1	76.7	75.9	76.2	75.9	77.8	77.0	77.3	76.6
		P	<.001	<.001	<.001	<.001	<.001	<.001	<.001	<.001	<.001	<.001	<.001	<.001
	eHEALS Group 2 vs base[c]	beta	0.21	0.21	0.21	0.2	0.11	0.11	-0.35	-0.35	-0.19	-0.18	-0.26	-0.26
		P	.57	.57	.53	.54	.73	.74	.38	.38	.56	.57	.38	.39
	eHEALS Group 3 vs base	beta	-0.22	-0.18	-0.42	-0.32	-0.44	-0.32	-0.43	-0.39	-0.62	-0.52	-0.65	-0.52
		P	.49	.55	.24	.34	.24	.36	.14	.16	.05	.08	.07	.10
	eHEALS Group 4 vs base	beta	-0.02	-0.05	-0.03	-0.06	-0.03	-0.06	-0.24	-0.26	-0.24	-0.27	-0.24	-0.27
		P	.95	.91	.93	.88	.93	.88	.47	.48	.46	.47	.46	.47

Logistic regression, the constant and coefficients of the following variables were not displayed: Age group, Education, Gender, Income, Paid employment, Family status, Residence, Self-perceived health, Activity limitations, Chronic morbidity, Setting, HCP type, Regular HCP

[a] Missed visit due to travel burden

[b] Likelihood ratio; omnibus test for independence, current model versus null model

[c] base: eHEALS Group 1

Check F dfs

Visit[a]		Scenario	#1	#2	#3	#4	#5	#6	#7	#8	#9	#10	#11	#12
	Sample	N	498	498	498	498	498	498	498	498	498	498	498	498
	LR test[b]	$Chi^2_{(28)}$	63.9	63.8	63.9	63.9	63.7	63.7	63.8	63.6	63.6	63.7	63.4	63.4
		P	<.001	<.001	<.001	<.001	<.001	<.001	<.001	<.001	<.001	<.001	<.001	<.001
	eHEALS Group 2 vs base[c]	beta	-0.18	-0.18	-0.38	-0.39	-0.3	-0.3	-0.04	-0.04	-0.33	-0.33	-0.22	-0.22
		P	.66	.65	.30	.30	.39	.39	.93	.93	.37	.37	.51	.51
	eHEALS Group 3 vs base	beta	-0.36	-0.33	-0.22	-0.21	-0.31	-0.27	-0.29	-0.26	-0.15	-0.14	-0.24	-0.2
		P	.29	.32	.54	.55	.44	.47	.36	.41	.66	.68	.53	.57
	eHEALS Group 4 vs base	beta	-0.24	-0.29	-0.25	-0.29	-0.25	-0.29	-0.16	-0.21	-0.17	-0.21	-0.17	-0.21
		P	.54	.51	.53	.51	.53	.51	.66	.61	.65	.61	.65	.61

Logistic regression, the constant and coefficients of the following variables were not displayed: Age group, Education, Gender, Income, Paid employment, Family status, Residence, Self-perceived health, Activity limitations, Chronic morbidity, Setting, HCP type, Regular HCP

[a] Missed visit due to cost burden

[b] Likelihood ratio; omnibus test for independence, current model versus null model

[c] base: eHEALS Group 1

Intervention[a]	Scenario	#1	#2	#3	#4	#5	#6	#7	#8	#9	#10	#11	#12
Sample	N	501	501	501	501	501	501	501	501	501	501	501	501
LR test[b]	$Chi^2_{(28)}$	47.6	46.6	46.7	45.8	47.9	46.3	47.3	46.3	46.5	45.5	47.6	46.1
	P	.01	.02	.01	.02	.01	.02	.01	.02	.02	.02	.01	.02
eHEALS Group 2 vs base[c]	beta	-0.28	-0.29	-0.46	-0.46	-0.42	-0.43	-0.19	-0.19	-0.42	-0.42	-0.38	-0.38
	P	.49	.48	.23	.22	.24	.23	.67	.67	.27	.27	.29	.29
eHEALS Group 3 vs base	beta	-0.82	-0.71	-0.82	-0.68	-1.03	-0.79	-0.75	-0.64	-0.75	-0.61	-0.97	-0.71
	P	.03	.04	.04	.07	.02	.049	.03	.05	.05	.08	.03	.06
eHEALS Group 4 vs base	beta	-0.14	-0.1	-0.15	-0.11	-0.15	-0.11	-0.06	-0.02	-0.07	-0.02	-0.07	-0.03
	P	.72	.82	.70	.80	.70	.8	.86	.97	.85	.96	.84	.95

Logistic regression, the constant and coefficients of the following variables were not displayed: Age group, Education, Gender, Income, Paid employment, Family status, Residence, Self-perceived health, Activity limitations, Chronic morbidity, Setting, HCP type, Regular HCP

[a] Missed intervention due to cost burden

[b] Likelihood ratio; omnibus test for independence, current model versus null model

[c] base: eHEALS Group 1

Medication[a]	Scenario	#1	#2	#3	#4	#5	#6	#7	#8	#9	#10	#11	#12
Sample	N	499	499	499	499	499	499	499	499	499	499	499	499
LR test[b]	$Chi^2_{(28)}$	92.7	92.5	93.5	94.2	93.1	93.8	92.7	92.5	93.5	94.1	93.1	93.7
	P	<.001	<.001	<.001	<.001	<.001	<.001	<.001	<.001	<.001	<.001	<.001	<.001
eHEALS Group 2 vs base[c]	beta	0.05	0.07	0.13	0.14	0.07	0.08	0.00	0.00	0.11	0.11	0.03	0.03
	P	.89	.85	.70	.68	.83	.81	.99	.99	.74	.74	.91	.91
eHEALS Group 3 vs base	beta	-0.04	-0.16	-0.17	-0.31	-0.15	-0.34	-0.06	-0.19	-0.19	-0.35	-0.18	-0.37
	P	.91	.61	.64	.36	.69	.34	.83	.51	.56	.27	.62	.27
eHEALS Group 4 vs base	beta	-0.28	0.03	-0.28	0.02	-0.28	0.01	-0.3	0.00	-0.31	-0.01	-0.31	-0.02
	P	.47	.94	.47	.96	.46	.97	.39	.99	.38	.97	.38	.96

Logistic regression, the constant and coefficients of the following variables were not displayed: Age group, Education, Gender, Income, Paid employment, Family status, Residence, Self-perceived health, Activity limitations, Chronic morbidity, Setting, HCP type, Regular HCP

[a] Missed medication due to cost burden

[b] Likelihood ratio; omnibus test for independence, current model versus null model

[c] base: eHEALS Group 1

oWP[a]		Scenario	#1	#2	#3	#4	#5	#6	#7	#8	#9	#10	#11	#12
	Sample	N	505	505	505	505	505	505	505	505	505	505	505	505
	LR test[b]	$Chi^2_{(28)}$	53.9	54.0	51.7	51.8	51.9	52.0	53.6	53.7	52.2	52.3	52.4	52.5
		P	.002	.002	.004	.004	.004	.004	.002	.002	.004	.004	.003	.003
	eHEALS Group 2 vs base[c]	beta	-0.22	-0.22	0.05	0.05	0.06	0.06	-0.07	-0.07	0.24	0.24	0.23	0.23
		P	.57	.57	.87	.87	.85	.85	.87	.87	.45	.46	.46	.46
	eHEALS Group 3 vs base	beta	0.32	0.31	0.26	0.27	0.31	0.31	0.4	0.39	0.34	0.35	0.39	0.39
		P	.31	.30	.43	.40	.37	.35	.16	.15	.27	.23	.23	.20
	eHEALS Group 4 vs base	beta	0.37	0.41	0.38	0.43	0.38	0.43	0.46	0.5	0.46	0.5	0.46	0.5
		P	.29	.28	.27	.26	.27	.26	.15	.16	.15	.16	.15	.16

Logistic regression, the constant and coefficients of the following variables were not displayed: Age group, Education, Gender, Income, Paid employment, Family status, Residence, Self-perceived health, Activity limitations, Chronic morbidity, Setting, HCP type, Regular HCP

[a] Office waiting time was a problem

[b] Likelihood ratio; omnibus test for independence, current model versus null model

[c] base: eHEALS Group 1

aWP[a]		Scenario	#1	#2	#3	#4	#5	#6	#7	#8	#9	#10	#11	#12
	Sample	N	502	502	502	502	502	502	502	502	502	502	502	502
	LR test[b]	$Chi^2_{(27)}$	63.7	65.1	63.6	64.7	63.5	64.8	63.8	65.1	63.8	66.0	63.7	65.0
		P	<.001	<.001	<.001	<.001	<.001	<.001	<.001	<.001	<.001	<.001	<.001	<.001
	eHEALS Group 2 vs base[c]	beta	-0.3	-0.29	-0.44	-0.44	-0.42	-0.41	-0.33	-0.33	-0.48	-0.48	-0.43	-0.43
		P	.48	.49	.25	.26	.26	.27	.47	.47	.22	.21	.23	.23
	eHEALS Group 3 vs base	beta	-0.47	-0.55	-0.38	-0.51	-0.4	-0.56	-0.43	-0.52	-0.34	-0.47	-0.37	-0.53
		P	.20	.12	.33	.18	.34	.16	.21	.12	.35	.18	.36	.16
	eHEALS Group 4 vs base	beta	-0.37	-0.06	-0.37	-0.06	-0.37	-0.06	-0.33	-0.02	-0.32	-0.02	-0.33	-0.02
		P	.38	.90	.38	.89	.38	.89	.41	.97	.41	.97	.41	.96

Logistic regression, the constant and coefficients of the following variables were not displayed: Age group, Education, Gender, Income, Paid employment, Family status, Residence, Self-perceived health, Activity limitations, Chronic morbidity, Setting, HCP type, Regular HCP

[a] Appointment waiting time was a problem

[b] Likelihood ratio; omnibus test for independence, current model versus null model

[c] base: eHEALS Group 1

Log oWT[a]		Scenario	#1	#2	#3	#4	#5	#6	#7	#8	#9	#10	#11	#12
	Sample	N	502	502	502	502	502	502	502	502	502	502	502	502
	LR test[b]	$F_{(28,473)}$	2.50	2.51	2.48	2.50	2.48	2.50	2.49	2.51	2.49	2.51	2.49	2.51
		P	<.001	<.001	<.001	<.001	<.001	<.001	<.001	<.001	<.001	<.001	<.001	<.001
	eHEALS Group 2 vs base[c]	beta	-0.08	-0.08	-0.02	-0.02	-0.01	-0.01	0.04	0.04	0.09	0.09	0.08	0.08
		P	.63	.64	.91	.91	.92	.93	.80	.80	.54	.54	.55	.55
	eHEALS Group 3 vs base	beta	0.02	0.01	-0.01	-0.01	-0.01	-0.02	0.06	0.06	0.04	0.04	0.03	0.03
		P	.91	.94	.94	.93	.93	.92	.60	.62	.78	.78	.81	.80
	eHEALS Group 4 vs base	beta	0.07	0.13	0.08	0.13	0.08	0.13	0.13	0.18	0.13	0.18	0.13	0.18
		P	.63	.46	.62	.45	.62	.45	.38	.27	.38	.27	.38	.27

Ordinary least squares (OLS) regression, the constant and coefficients of the following variables were not displayed: Age group, Education, Gender, Income, Paid employment, Family status, Residence, Self-perceived health, Activity limitations, Chronic morbidity, Setting, HCP type, Regular HCP

[a] Log-office waiting time

[b] Likelihood ratio; omnibus test for independence, current model versus null model

[c] base: eHEALS Group 1

Log aWT[a]	Scenario	#1	#2	#3	#4	#5	#6	#7	#8	#9	#10	#11	#12
Sample	N	492	492	492	492	492	492	492	492	492	492	492	492
LR test[b]	$F_{(28,455)}$	8.99	9.02	8.92	8.95	8.93	8.94	9.02	9.05	8.92	8.95	8.93	8.94
	P	<.001	<.001	<.001	<.001	<.001	<.001	<.001	<.001	<.001	<.001	<.001	<.001
eHEALS Group 2 vs base[c]	beta	0.15	0.16	0.02	0.02	-0.04	-0.03	0.24	0.24	0.03	0.03	-0.04	-0.04
	P	.51	.50	.92	.91	.86	.87	.34	.34	.89	.89	.82	.82
eHEALS Group 3 vs base	beta	-0.08	-0.09	-0.03	-0.05	0.05	0.00	-0.08	-0.09	-0.03	-0.05	0.05	0.00
	P	.69	.65	.88	.79	.83	.99	.66	.61	.87	.77	.81	.99
eHEALS Group 4 vs base	beta	0.03	0.12	0.03	0.11	0.03	0.11	0.02	0.11	0.02	0.11	0.03	0.11
	P	.90	.64	.91	.65	.91	.65	.91	.63	.91	.64	.90	.63

Ordinary least squares (OLS) regression, the constant and coefficients of the following variables were not displayed: Age group, Education, Gender, Income, Paid employment, Family status, Residence, Self-perceived health, Activity limitations, Chronic morbidity, Setting, HCP type, Regular HCP

[a] Log-appointment waiting time

[b] Likelihood ratio; omnibus test for independence, current model versus null model

[c] base: eHEALS Group 1

Square-root Unmet Medical Needs[a]	Scenario	#1	#2	#3	#4	#5	#6	#7	#8	#9	#10	#11	#12
Sample	N	484	484	484	484	484	484	484	484	484	484	484	484
LR test[b]	$F_{(28,455)}$	7.03	7.00	7.16	7.11	7.41	7.29	7.03	7.00	7.15	7.11	7.42	7.3
	P	<.001	<.001	<.001	<.001	<.001	<.001	<.001	<.001	<.001	<.001	<.001	<.001
eHEALS Group 2 vs base[c]	beta	-0.06	-0.06	-0.05	-0.05	-0.06	-0.06	-0.07	-0.07	-0.05	-0.05	-0.06	-0.06
	P	.51	.51	.57	.57	.48	.48	.49	.49	.58	.58	.47	.47
eHEALS Group 3 vs base	beta	-0.12	-0.12	-0.16	-0.15	-0.17	-0.16	-0.11	-0.11	-0.15	-0.14	-0.16	-0.15
	P	.14	.13	.07	.07	.05	.06	.13	.12	.06	.06	.04	.049
eHEALS Group 4 vs base	beta	-0.09	-0.06	-0.09	-0.06	-0.09	-0.06	-0.08	-0.05	-0.08	-0.05	-0.08	-0.05
	P	.36	.56	.36	.55	.36	.55	.38	.59	.38	.58	.37	.58

Ordinary least squares (OLS) regression with robust standard errors, the constant and coefficients of the following variables were not displayed: Age group, Education, Gender, Income, Paid employment, Family status, Residence, Self-perceived health, Activity limitations, Chronic morbidity, Setting, HCP type, Regular HCP

[a] Unmet Medical Needs Score: the number of aspects that contributed to the experience of an unmet need (missed visit due to travel burden; missed visit due to cost burden; missed intervention due to cost burden and; missed medication due to cost burden)

[b] Likelihood ratio; omnibus test for independence, current model versus null model

[c] base: eHEALS Group 1

**Any unmet need**[a]		**Scenario**	**#1**	**#2**	**#3**	**#4**	**#5**	**#6**	**#7**	**#8**	**#9**	**#10**	**#11**	**#12**
	Sample	N	481	481	481	481	481	481	481	481	481	481	481	481
	LR test[b]	$Chi^2_{(27)}$	111.1	111.1	114.0	114.3	114.1	114.3	111.6	111.6	114.1	114.3	114.3	114.5
		P	<.001	<.001	<.001	<.001	<.001	<.001	<.001	<.001	<.001	<.001	<.001	<.001
	eHEALS Group 2 vs base[c]	beta	-0.11	-0.11	-0.01	-0.01	-0.06	-0.05	-0.28	-0.27	-0.08	-0.08	-0.13	-0.13
		P	.75	.75	.98	.99	.85	.85	.46	.46	.79	.79	.64	.64
	eHEALS Group 3 vs base	beta	-0.33	-0.36	-0.54	-0.53	-0.62	-0.6	-0.36	-0.39	-0.57	-0.57	-0.65	-0.63
		P	.26	.21	.09	.08	.07	.06	.18	.13	.05	.04	.04	.03
	eHEALS Group 4 vs base	beta	-0.38	-0.3	-0.38	-0.3	-0.38	-0.3	-0.41	-0.33	-0.41	-0.34	-0.42	-0.34
		P	.26	.44	.26	.43	.26	.42	.19	.35	.19	.35	.19	.34

Logistic regression, the constant and coefficients of the following variables were not displayed: Age group, Education, Gender, Income, Paid employment, Family status, Residence, Self-perceived health, Activity limitations, Chronic morbidity, Setting, HCP type, Regular HCP

[a] Any Unmet Medical Need: any unmet need experienced (missed visit due to travel burden; missed visit due to cost burden; missed intervention due to cost burden and; missed medication due to cost burden)

[b] Likelihood ratio; omnibus test for independence, current model versus null model

[c] base: eHEALS Group 1

**Any WP**[a]		**Scenario**	**#1**	**#2**	**#3**	**#4**	**#5**	**#6**	**#7**	**#8**	**#9**	**#10**	**#11**	**#12**
	Sample	N	505	505	505	505	505	505	505	505	505	505	505	505
	LR test[b]	$Chi^2_{(28)}$	75.1	75.0	74.8	74.7	74.4	74.3	75.1	75.0	74.8	74.7	74.4	74.3
		P	<.001	<.001	<.001	<.001	<.001	<.001	<.001	<.001	<.001	<.001	<.001	<.001
	eHEALS Group 2	beta	-0.16	-0.16	-0.06	-0.06	0.00	0.00	-0.16	-0.16	-0.02	-0.02	0.05	0.05
	vs base[c]	*P*	.65	.65	.84	.85	.99	.99	.67	.67	.94	.94	.87	.87
	eHEALS Group 3	beta	0.17	0.15	0.21	0.17	0.19	0.15	0.20	0.18	0.23	0.20	0.22	0.18
	vs base	*P*	.56	.60	.50	.56	.56	.62	.46	.49	.42	.47	.48	.53
	eHEALS Group 4	beta	0.04	0.04	0.05	0.05	0.05	0.05	0.07	0.08	0.07	0.08	0.07	0.08
	vs base	*P*	.91	.90	.89	.88	.89	.88	.82	.83	.81	.81	.81	.82

Logistic regression, the constant and coefficients of the following variables were not displayed: Age group, Education, Gender, Income, Paid employment, Family status, Residence, Self-perceived health, Activity limitations, Chronic morbidity, Setting, HCP type, Regular HCP

[a] Any Waiting Problem: the office waiting time or appointment waiting time was a problem

[b] Likelihood ratio; omnibus test for independence, current model versus null model

[c] base: eHEALS Group 1
