# Supplementary material for: Exploring eHealth Literacy and Patient-Reported Experiences With Outpatient Care in the Hungarian General Adult Population: Cross-Sectional Study
Source: J Med Internet Res. 2020 Aug 11;22(8):e19013. doi: 10.2196/19013 (PMC7448194; doi:10.2196/19013)
Supplement: Multimedia Appendix 7 [file jmir_v22i8e19013_app7.pdf]

Zrubka Z, Brito Fernandes O, Baji P, Hajdu O, Kovács L, Kringos D; Klazinga N, Gulácsi L, Brodszky V, Rencz F, Péntek M. eHealth Literacy and Patient-Reported Experiences with Outpatient Care in the Hungarian General Adult Population: A Cross-Sectional Survey Study. *J Med Internet Res*. 2020

Multimedia Appendix 7

Regression analyses of unmet medical needs for those respondents, who had health problem over the past 12 months

	**Travel[a]**		**Visit[b]**		**Intervention[c]**		**Medication[d]**		**Square root Unmet Medical Needs Score[e]**		**Any Unmet Medical Needs[f]**	
Model	Logistic		Logistic		Logistic		Logistic		Robust[p]		Logistic	
	Beta	*P*	Beta	*P*	Beta	*P*	Beta	*P*	Beta	*P*	Beta	*P*
**eHEALS[g]**												
2nd quartile	-0.08	.78	-0.33	.29	-0.34	.32	0.03	.91	-0.03	.68	-0.03	.92
3rd quartile	-0.22	.42	-0.19	.51	-0.35	.28	-0.04	.88	-0.08	.27	-0.36	.16
4th quartile	-0.06	.84	-0.10	.74	0.14	.66	-0.15	.63	-0.04	.65	-0.31	.26
**Age group[h]**												
25-44 years old	-0.24	.48	-0.09	.82	0.73	.14	-0.33	.38	-0.17	.1	-0.87	.02
45-64 years old	-0.74	.048	-0.95	.02	-0.08	.88	-0.59	.15	-0.37	<.001	-1.54	<.001
65+ years old	-1.23	.004	-1.23	.008	0.09	.87	-0.91	.045	-0.43	<.001	-1.59	<.001
**Education[i]**												
Secondary	0.05	.82	-0.07	.78	-0.09	.77	0.25	.33	0.01	.89	0.03	.89
Tertiary	-0.66	.03	-0.58	.07	-0.39	.25	-1.03	.002	-0.21	.005	-0.76	.005
**Gender**												
Male	-0.32	.13	-0.32	.16	-0.04	.87	-0.43	.06	-0.11	.07	-0.35	.08
**Income[j]**												
2nd quintile	-0.04	.88	-0.02	.94	0.34	.32	0.24	.42	0.01	.89	-0.13	.65
3rd quintile	-0.64	.11	-0.45	.29	-0.28	.55	0.13	.74	-0.13	.2	-0.52	.15
4th quintile	-0.18	.58	-0.10	.79	-0.19	.64	-0.03	.93	-0.07	.48	-0.33	.3
5th quintile	-0.35	.25	-0.38	.26	-0.34	.36	-0.58	.09	-0.19	.02	-0.76	.008
**Paid employment**												
Yes	-0.02	.93	0.15	.57	0.10	.73	-0.02	.94	-0.03	.7	-0.25	.29
**Family status**												
Married / domestic partnership	0.33	.1	0.09	.7	0.37	.13	-	1.	0.05	.39	0.14	.47
**Residence[k]**												
City	-0.34	.19	-0.50	.06	0.46	.17	-0.20	.49	-0.10	.12	-0.49	.04
Village	-0.34	.27	-0.42	.2	0.09	.83	-0.30	.38	-0.10	.25	-0.33	.26
**Self-perceived health[l]**												
Very bad	1.80	.16	0.28	.81	1.65	.17	0.91	.49	0.71	.002	-	-
Bad	1.50	.005	0.37	.5	1.17	.07	0.03	.95	0.41	.009	1.62	.002
Fair	0.65	.14	-0.10	.82	0.67	.21	0.25	.59	0.19	.11	0.88	.03
Good	0.29	.48	-0.18	.65	0.09	.86	-0.58	.18	0.05	.61	0.43	.24
**GALI[m]**												
Limited but not severely	0.41	.08	0.47	.06	0.23	.41	0.90	<.001	0.16	.01	0.46	.04
Severely limited	0.79	.06	0.75	.09	0.50	.27	1.60	<.001	0.33	.03	0.86	.046
**Chronic morbidity**												
Yes	0.19	.44	0.67	.02	0.37	.25	0.42	.15	0.19	.008	0.56	.02
**Constant**	-0.66	.28	-0.58	.36	-3.01	<.001	-0.84	.19	0.81	<.001	0.83	.15
n	626		626		627		631		605		601	
LR test Chi-square (24)	81.1	<.001	59.0	<.001	47.6	.09	107.3	<.001				
LR test F(24,580)									8.47	<.001		
LR test Chi-square (23)											124.7	<.001
R^2									0.20			
Ramsey RESET test F(3;577)									2.40	.07		
GOF[o] test Chi-square (580)	589.7	.38			610.0	.19						
GOF[o] test Chi-square (579)			604.9	.22								
GOF[o] test Chi-square (586)							593.6	.40				
GOF test Chi-square (558)											579.1	.26

[a]Office waiting time was a problem
[b]Appointment waiting time was a problem
[c]Log-office waiting time
[d]Log- appointment waiting time

[e]Unmet Medical Needs Score: the number of aspects that contributed to the experience of an unmet need (missed visit due to travel burden; missed visit due to cost burden; missed intervention due to cost burden and; missed medication due to cost burden)
[f]Any Unmet Medical Need: any unmet need experienced (missed visit due to travel burden; missed visit due to cost burden; missed intervention due to cost burden
[g]Base: 1st quartile
[h]Base: 18-24 years old
[i]Base: Primary
[j]Base: 1st quintile
[k]Base: Capital
[l]Base: Very goodr
[m]Base: Not limited
[n]Likelihood ratio; omnibus test for independence, current model versus null model
[o]Goodness of fit, Hosmer-Lemeshow test
[p]Ordinary least squares regression with robust standard errors
